# Off-the-shelf dual CAR-iNKT cell immunotherapy eradicates medullary and leptomeningeal high-risk *KMT2A*-rearranged leukemia^∗^

**DOI:** 10.1182/blood.2025029302

**Published:** 2025-09-04

**Authors:** Hongwei Ren, Natalina Elliott, Bryan Lye, Mohammad Umer Sharif Shohan, Joe W. Cross, Lucy Field, Kanagaraju Ponnusamy, Siobhan Rice, Thomas Jackson, Ilia Leontari, Nouhad El Ouazzani, Rebecca Thomas, Sarah Inglott, Jack Bartram, Owen Smith, Jonathan Bond, Irene A. G. Roberts, Christina Halsey, Rachael Bashford-Rogers, Thomas A. Milne, Anindita Roy, Anastasios Karadimitris

**Affiliations:** 1 Centre for Haematology, Department of Immunology and Inflammation, Imperial College London, London, United Kingdom; 2 Department of Paediatrics, University of Oxford, Oxford, United Kingdom; 3 Department of Biochemistry, University of Oxford, Oxford, United Kingdom; 4 MRC Molecular Haematology Unit, MRC Weatherall Institute of Molecular Medicine, University of Oxford, Oxford, United Kingdom; 5 Department of Haematology, Great Ormond Street Hospital, London, United Kingdom; 6 School of Medicine, Trinity College, University of Dublin, Dublin, Ireland; 7 National Children's Cancer Service, Children's Health Ireland at Crumlin, Dublin, Ireland; 8 Systems Biology Ireland, School of Medicine, University College Dublin, Dublin, Ireland; 9 Wolfson Wohl Cancer Research Centre, College of Medical Veterinary and Life Sciences, School of Cancer Sciences, University of Glasgow, Glasgow, United Kingdom; 10 Cancer Research UK Oxford Centre, University of Oxford, Oxford, United Kingdom; 11 Department of Haematology, Hammersmith Hospital, Imperial College Healthcare NHS Trust, London, United Kingdom

## Abstract

Current therapies, including autologous chimeric antigen receptor (CAR) T-cell immunotherapy, fail to cure half of infants with *KMT2A*-rearranged acute lymphoblastic leukemia (KMT2Ar-ALL), a disease characterized by frequent central nervous system involvement, poor treatment response, early relapse, and lineage switching. More effective treatment strategies, including the availability of off-the-shelf immunotherapies, is particularly relevant in infants. PROM1/CD133 is a direct target of KMT2A-fusion oncoproteins and is expressed on leukemic cells. Allogeneic invariant natural killer T (iNKT) cells, “innately” more powerful effectors than T cells, can be deployed off-the-shelf without risk of acute graft-versus-host disease. Here, we equip iNKT cells with CD19- and/or CD133-targeting CARs, and investigate their antileukemia activity against KMT2Ar-ALL in relevant in vitro and in vivo models. Compared with monospecific counterparts and dual, bispecific CAR T cells, bispecific CD19-CD133 CAR-iNKT cells have a more potent antileukemia activity, effectively targeting both CAR antigen–high and –low leukemia. Bispecific CAR-iNKT cells eradicate medullary and, notably, leptomeningeal leukemia, and induce sustained remissions without discernible hematologic toxicity. Mechanistically, the more potent antileukemia effect of CAR-iNKT cells over CAR T cells is mediated by a pronounced CAR-dependent and CAR antigen–dependent upregulation of the innate activating receptor NKG2D on CAR-iNKT cells, and its engagement by its corresponding ligands on KMT2Ar-ALL cells. This ensures effective leukemia targeting even with downregulation of CD133 or CD19. Thus, by engaging with 2 different types of leukemia-associated antigens, that is, CAR antigens and NKG2D ligands, CAR-iNKT cells provide a powerful platform for the treatment of KMT2Ar-ALL. This approach can be readily adapted for other high-risk malignancies, including those with otherwise difficult to target leptomeningeal involvement.

## Key Points

• Off-the-shelf bispecific CD133-CD19 CAR-iNKT cells eradicate CAR antigen–high and –low medullary and leptomeningeal KMT2Ar ALL.
• CAR– and CAR antigen–dependent upregulation of NKG2D underpins the higher potency of bispecific CAR-iNKT cells over CAR T-cell counterparts.

## Visual Abstract

**Figure:**
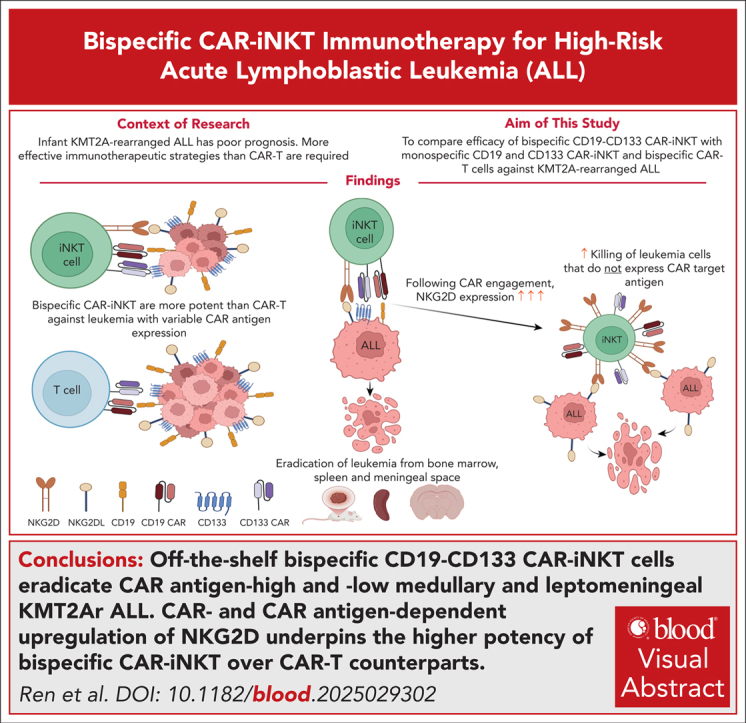

## Teaser

Invariant natural killer T (iNKT) cells are innate lymphocytes that display potent antitumor effects but do not induce graft-versus-host disease. Ren and colleagues report that, in these allogeneic cells, there is upregulation of the activating receptor NKG2D, which also engages with ligands expressed on leukemia cells to enhance chimeric antigen receptor (CAR)–instigated killing. Consequently, bispecific CAR-iNKT cells targeting CD19 and CD133 exhibit superior antileukemic activity compared with conventional CAR T cells in preclinical models of *KMT2A*-rearranged leukemia, prompting clinical development of this class of product.

## Introduction

Acute leukemias initiated by rearrangement and fusion of the *KMT2A* (*MLL*) gene to a variety of partners are among the hematologic malignancies with the worst prognoses. *KMT2A*-rearranged (KMT2Ar) B-cell acute lymphoblastic leukemia (B-ALL) is the most common form of infant ALL (80%),^1^ and comprises a smaller fraction of childhood (3%) and adult (10%) ALLs as well.^2^^,^^3^ Compared with the >90% survival of childhood B-ALL, event-free survival of infants and children with KMT2Ar B-ALL is 38% and 65%, respectively, mainly due to chemoresistance and relapse.^4^^,^^5^ Recent use of bispecific CD19-targeting T-cell engagers appears to improve survival, while CD19 chimeric antigen receptor (CAR) T-cell immunotherapy offers promise of rescuing a subset of patients with relapsed disease who would otherwise have a dismal prognosis.^6^^,^^7^ Nevertheless, after CD19 CAR T-cell therapy, one-third of patients relapse with either CD19^+^ or CD19^−^ disease, including lineage-switched disease,^8^^,^^9^ which is associated with partial or complete loss of CD19 expression,^8^^,^^10^ and escape from CD19 CAR T-cell control.^8^^,^^11^ Treatment failure in KMT2Ar-ALL is also linked to the inability of current therapeutic approaches to effectively eradicate central nervous system disease, usually manifesting as leptomeningeal infiltration.^12^

We previously showed that *PROM1* (CD133) is a direct target of leukemic KMT2A fusion proteins, and that KMT2Ar-ALL typically expresses PROM1/CD133.^13^^,^^14^ Therefore, dual targeting of CD19 and CD133 provides a rational approach to enhance antileukemic activity and limit immune escape. In support of this, a preclinical approach involving a tandem CD19-CD133 CAR has been described,^15^ although direct comparison of the bispecific approach with CD133 CAR T cells and in vivo study of potential hematologic toxicity were not performed. This is particularly important as CD133 is also expressed on normal hematopoietic stem and progenitor cells (HSPCs).^16^, ^17^, ^18^ In addition, previous preclinical data suggest that tandem CARs may be less effective than bispecific designs,^19^^,^^20^ possibly due to steric hindrance mechanisms that interfere with high-affinity binding of each CAR to their target antigens.

As well as optimal CAR design and target selection, effective immunotherapy would also benefit from the cooperative activity of powerful and multifunctional effector cells. In this regard, we and others have developed the CD1d-restricted, glycolipid-reactive invariant natural killer T (iNKT) cells.^21^, ^22^, ^23^ iNKT cell–based immunotherapy has the major advantage, particularly for pediatric malignancies, of providing a versatile off-the-shelf platform^24^, ^25^, ^26^, ^27^ that not only lacks the risk of inciting acute graft-versus-host disease (aGVHD),^28^^,^^29^ but also has inherent antitumor activity against CD1d-expressing tumors that may complement that of the CAR modules. Underscoring this point, we previously showed that CAR-iNKT cells are more effective against CD1d-expressing lymphoma than their CAR T-cell counterparts,^26^^,^^30^ and KMT2Ar ProB ALL has been previously reported to express CD1d.

Here, we describe the development of iNKT cells equipped with a dual, bispecific CAR against CD19 and CD133 as a potential off-the-shelf therapy for infant ALL, and investigate their activity against high-risk medullary and meningeal KMT2Ar-ALL.

## Methods

### Samples

Peripheral blood mononuclear cells obtained from healthy donors were isolated by density gradient centrifugation, and used as a source of CD3^+^ iNKT and T cells for CAR engineering.

Cell lines were available in the laboratory of T.A.M. SEM cells expressing Luc-DsRed were generated using a retroviral vector. CRISPR/Cas9 gene editing was used to generate SEM cells lacking expression of antigens of interest.

### Animals

Experimental animals were 8- to 12-week-old NOD.Cg-Prkdcscid Il2rgtm1Wjl/SzJ mice (NSG; Jackson Laboratories) or 7- to 9-week-old NSGS mice (see the supplemental Methods for experimental details, available on the *Blood* website).

### Generation of CAR-iNKT cells and CAR T cells

Generation of CAR-iNKT cells and CAR T cells was performed as previously described.^26^ Briefly, for the anti-CD19 single-target CAR, the FMC63 antibody clone, along with partial intracellular domain of CD8α (amino acids 128-210 of CD8α), CD28 costimulatory domain, and CD3ζ activation domain were used. For the anti-CD133 single-target CAR, the AC133 antibody clone, incorporating the same CD8α components as the anti-CD19 CAR was used, but coupled with the 4-1BB costimulatory domain and CD3ζ activation domain. The bispecific CD19-CD133 dual CAR was engineered by linking the aforementioned single-target CARs via a T2A peptide sequence. T cells or iNKT cells were transduced with concentrated CAR lentivirus.

### In vitro assays

Cytotoxicity assays and intracellular cytokine expression assays were performed as previously described.^26^^,^^27^

### sc Assays

CAR-iNKT cells were enriched by magnetic bead selection from the bone marrow (BM) of mice that either did or did not receive SEM cells. In addition, 30 000 preinfusion CAR-iNKT cells from the same donor were also fluorescence-activated cell sorted for single-cell (sc) analysis. Cells were immediately processed using the T-cell receptor (TCR)/RNA sequencing protocol (10× genomics CG000331 Rev C).

### Additional methods

Further methods are provided in the supplementary Material.

Appropriate ethical approval was in place for all human samples. Experiments were performed under the Animal (Scientific Procedures) Act 1986 with institutional approval by the local ethical review body and Home Office license (Animal Welfare and Ethical Review Body, Imperial College London, London, United Kingdom; Clinical Medicine Animal Welfare and Ethical Review Body, University of Oxford, Oxford, United Kingdom).

## Results

### Enhanced antileukemic activity of bispecific CAR-iNKT cells

Immunophenotypic analysis of primary blast cells from patients with KMT2Ar ALL, in agreement with our previous study,^14^ showed coexpression of CD19 and CD133, with a 67% median level of CD133 coexpression (range 0%-100%; supplemental Figure 1A-B). Contrary to previous reports,^31^ we found that most patient samples we tested were negative for CD1d expression (supplemental Figure 1A,C). Similarly, ALL blasts isolated from the BM of our recently described KMT2Ar infant ALL (^CRISPR^KMT2A-AFF1 ALL)^32^ demonstrated variable expression of CD133, and minimal expression of CD1d on CD19^+^ blasts (supplemental Figure 1A-B). CD133 but not CD19 is expressed on normal primitive fetal hematopoietic stem cell/multipotent progenitors (Lin^–^CD34^+^CD38^–^), while fetal B-progenitors (CD34^+^CD19^+^) express CD19 but not CD133, confirming that CD133/CD19 coexpression is leukemia-specific (supplemental Figure 1D).

**Figure 1.**
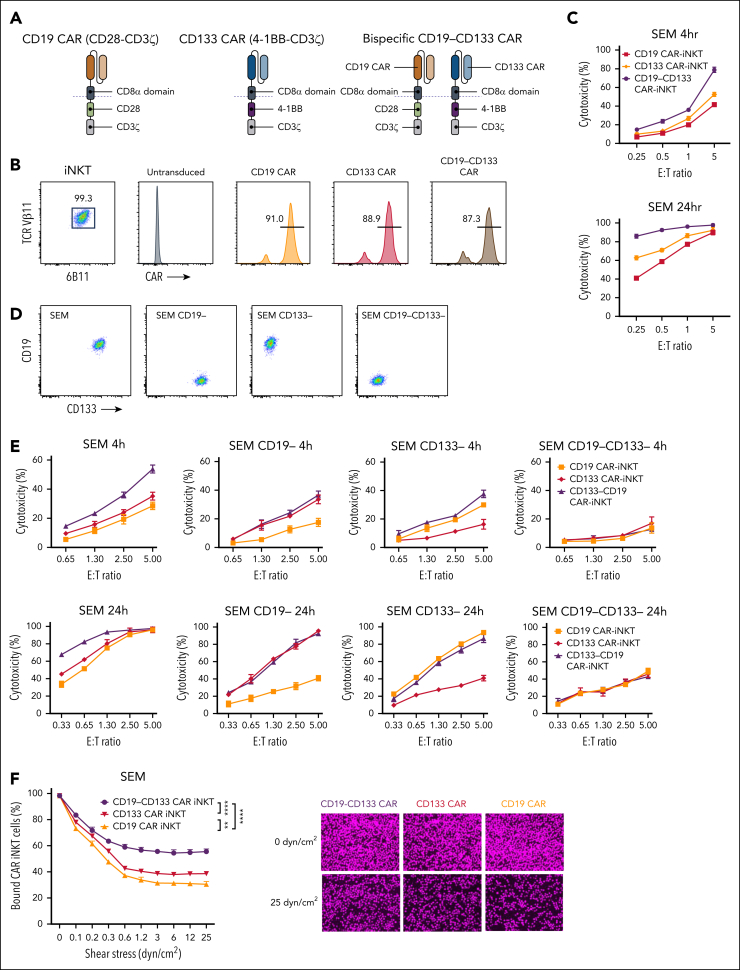
**Design and in vitro validation of bispecific CD19-CD133 CAR-iNKT cells.** (A) Design of CD19 and CD133 monospecific and CD19-CD133 bispecific CARs. (B) Flow cytometric analysis of highly pure, expanded iNKT cells, gated on CD3 and coexpressing invariant TCRVα24 (identified with the 6B11 mAb) and TCRVβ11, expressing the indicated CARs, as assessed by L-protein staining. (C) Cytotoxicity assay of indicated mono- and bispecific CAR-iNKT cells against SEM cells at 4 and 24 hours. Error bars represent standard deviation of triplicate assays. (D) Flow cytometric analysis of CD19 and CD133 expression in parental and gene-edited SEM leukemia cells. (E) Cytotoxicity assay of mono- and bispecific CAR-iNKT cells against the target cells shown in panel D. (F) Flow chamber avidity assay of indicated CAR-iNKT cells over a range of pressures (left), and representative images of CAR-iNKT cells bound to SEM cells at the beginning and end of the assay (right). Data are presented as the mean ± SEM of n = 3 experiments. ∗∗*P* < .01; ∗∗∗∗*P* < .0001, by 1-way analysis of variance (ANOVA).

Starting from 2 monoclonal antibody (mAb) clones, we generated monospecific second-generation 28z CD19 and 4-1BBz CD133 CARs, and the corresponding dual, bispecific CAR (henceforth, bispecific), such that each CAR would be expressed stoichiometrically and independently of each other in the same cell (Figure 1A). Using our previously described protocol,^26^^,^^27^ we generated peripheral blood-derived CAR-iNKT cells with all 3 CARs stably and similarly highly expressed (Figure 1B). The corresponding CAR-iNKT cells exhibited similar growth rates (supplemental Figure 2A). When tested against the KMT2A::AFF1 CD19^+^CD133^+^ B-ALL cell line SEM, bispecific CAR-iNKT cells were more cytotoxic than their monospecific counterparts in 4-hour and 24-hour cytotoxicity assays (Figure 1C). When tested against SEM cells gene-edited to lack expression of either or both CD19 and CD133, bispecific CAR-iNKT cells demonstrated the highest cytotoxic activity against CD19^+^CD133^+^ targets, and equivalent cytotoxicity as the monospecific CAR-iNKT cells against CD19^+^CD133^−^ or CD19^−^CD133^+^ targets (Figure 1D-E). This effect was also validated against RS4;11, another CD19^+^CD133^+^ KMT2Ar-ALL cell line (supplemental Figure 2B).

These findings confirm that both CD19 and CD133 CARs are specific and active as bispecific as well as monospecific CARs, with bispecific CAR-iNKT cells exerting a more robust antileukemia activity in vitro. Finally, we found that higher antileukemia cytotoxicity of bispecific CAR-iNKT cells is commensurate with higher avidity than their monospecific counterparts (Figure 1F).

### Bispecific CAR-iNKT cells outperform monospecific CAR-iNKT cells, and are active against low CAR antigen disease in vivo

**Figure 2.**
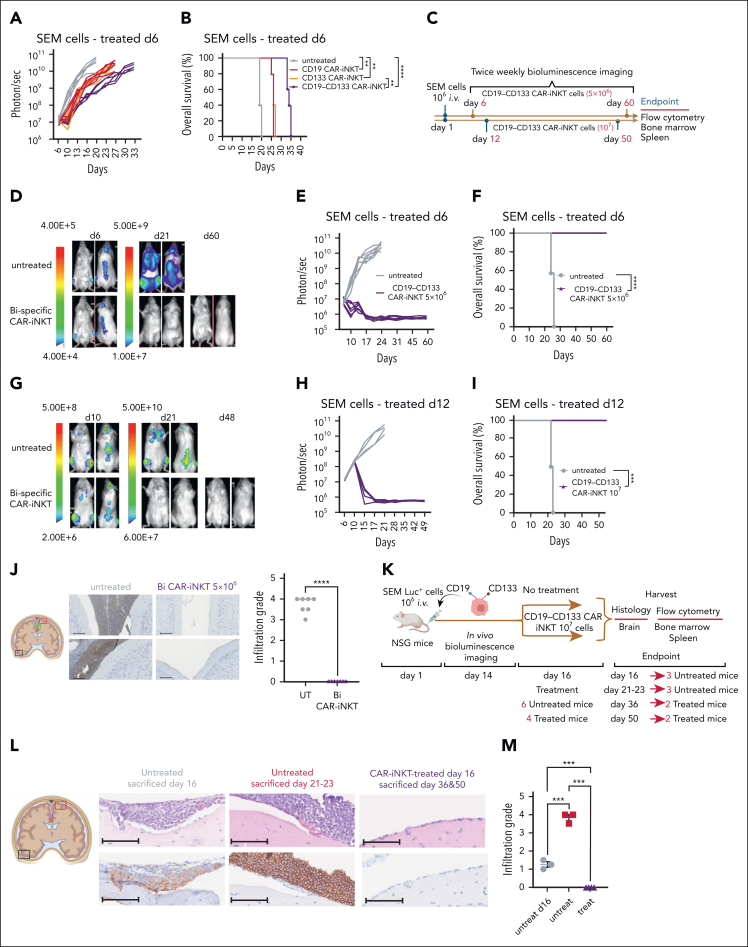
**Eradication of medullary and meningeal KMT2Ar-ALL by bispecific CAR-iNKT cells.** (A-B) Leukemia burden as assessed by bioluminescence imaging (BLI) and overall survival of SEM leukemia-bearing mice treated with 10^6^ mono- or bispecific CAR-iNKT cells on day 6 after leukemia transfer. (C) Schematic of experiments shown in panels D-I. (D-F) Representative BLI images (D), leukemia burden (E), and overall survival (F) in mice treated with 5 × 10^6^ bispecific CAR-iNKT cells 6 days after IV leukemia cell transfer (n = 7 mice per group). (G-I) Representative BLI images (G), leukemia burden (H), and overall survival (I) in mice treated with 10^7^ bispecific CAR-iNKT cells 12 days after IV leukemia cell transfer (n = 5 mice per group). (B, F, I) Kaplan-Meier curves were plotted for overall survival, with differences assessed by log-rank test (n = 5-7 mice per group). ∗∗*P* < .01; ∗∗∗*P* < .001; ∗∗∗∗*P* < .0001. (J) Left: Coronal sections of representative mouse brains and immunohistochemistry from end of experiment untreated control and bispecific CAR-iNKT cell 5 × 10^6^ treated mice (as shown in D-F) stained with antihuman CD19 mAb. Imaging region indicated by green box. Scale bar, 100 μm. Right: Cumulative meningeal infiltration grade scores. (K) Schematic of experiment for data shown in panels L-M. (L) Coronal sections of representative mouse brains from untreated mice euthanized on day 16, untreated mice euthanized at termination on days 21 to 23, and from day 16 treated mice euthanized either on day 36 or day 50. Top panels: hematoxylin and eosin staining; bottom panels: immunohistochemistry against human CD19. Day 16 area indicated by black box, and the other 2 groups by red box (left). Scale bar, 100 μm. (M) Cumulative meningeal infiltration grade scores in untreated mice euthanized on day 16, untreated mice euthanized at termination on days 21 to 23, and in day 16 treated mice euthanized either on day 36 or day 50. Mann-Whitney or 1-way ANOVA. ∗∗*P* < .01; ∗∗∗*P* < .001; ∗∗∗∗*P* < .0001. Bi, bispecific; UT, untreated.

To investigate the antileukemic potency of CAR-iNKT cells in vivo, we transferred a subtherapeutic dose of bispecific CAR-iNKT cells^33^ into mice engrafted with luciferase-dsRed-labeled SEM leukemia cells. In this model, untreated mice develop an aggressive form of universally fatal ALL with medullary and extramedullary (spleen, liver, and leptomeningeal) disease by days 23 to 25.^34^ First, using the parental CD19^+^CD133^+^ SEM cells, we confirmed the enhanced ability of bispecific CAR-iNKT cells to control leukemia and prolong overall survival when compared with each of the monospecific CAR-iNKT cells at the limiting dose of 10^6^ cells (Figure 2A-B).

Next, we used a SEM subline that after in vivo propagation displayed a spectrum of CD19 and CD133 coexpression, from high, low, to no expression of both markers (supplemental Figure 2C). Again, 10^6^ but not lower doses of bispecific CAR-iNKT cells delayed leukemia growth as assessed by bioluminescence imaging (BLI), and significantly prolonged survival (supplemental Figure 2D-E). Importantly, while all the residual leukemia cells expressed dsRed, they were CD19^–^CD133^–^ (supplemental Figure 2F), suggesting complete elimination of target cells coexpressing CD19 and CD133 even when expressed at low levels, and in line with the higher avidity of bispecific CAR-iNKT cells.

### Bispecific CAR-iNKT cells eradicate medullary and meningeal leukemia

To investigate whether bispecific CAR-iNKT cells can truly eradicate an established leukemia, we treated SEM leukemia-bearing mice with 5 × 10^6^ CAR-iNKT cells (Figure 2C-D,F) on day 6, or 10^7^ CAR-iNKT cells on day 12 (Figure 2C,G-I), when leukemia burden in the BM was at ∼2% and ∼15%, respectively (supplemental Figure 3A). In both experiments, leukemia burden as assessed by BLI rapidly declined to baseline, with 100% of animals surviving long term (Figure 2D-F,G-I). Furthermore, detailed analysis of the BM and spleen at termination showed lack of leukemia as well as effector cells (supplemental Figure 3B). Critically, while in untreated animals the meninges were heavily infiltrated by leukemia cells as identified by hematoxylin and eosin, and CD19 staining of brain sections (infiltration grade >3/5), leukemia cells were not identified in the meninges of CD19-CD133 CAR-iNKT cell–treated animals (Figure 2J).

Therefore, CD19-CD133 bispecific CAR-iNKT cells can eradicate leukemia from the BM and spleen, and protect from meningeal disease, thus resulting in deep, lasting remissions.

We then investigated whether bispecific CAR-iNKT cells can eradicate established meningeal leukemia using 3 leukemia-bearing groups (Figure 2K). The first group was untreated, and when assessed for meningeal disease on day 16 by detailed immunohistochemical analysis was found to have a mean leukemia meningeal infiltration grade of 1.25/5. A second untreated group using an end point of days 21 to 23 had an infiltration grade of 3.8/5 (Figure 2L-M). In a third group of animals treated with bispecific CAR-iNKT cells on day 16, consistent with meningeal leukemia eradication, histological, and immunohistochemical analysis of the brain either on day 36 or day 50 showed complete absence of any CD19^+^ cells (infiltration grade 0/5; Figure 2L-M). Of note, some meningeal stromal thickening was observed, likely representing areas of old leukemic infiltrates cleared by CAR-iNKT cell treatment (supplemental Figure 3C). There was no evidence of leukemia in the BM and spleen (supplemental Figure 3D-F). We conclude that bispecific CAR-iNKT cells outperform their monospecific counterparts, and at high doses display curative potential against medullary and extramedullary disease. Critically, they also display ability to eradicate and protect from meningeal leukemia.

### Bispecific CD19-CD133 CAR-iNKT cells eradicate primary KMT2Ar-ALL

**Figure 3.**
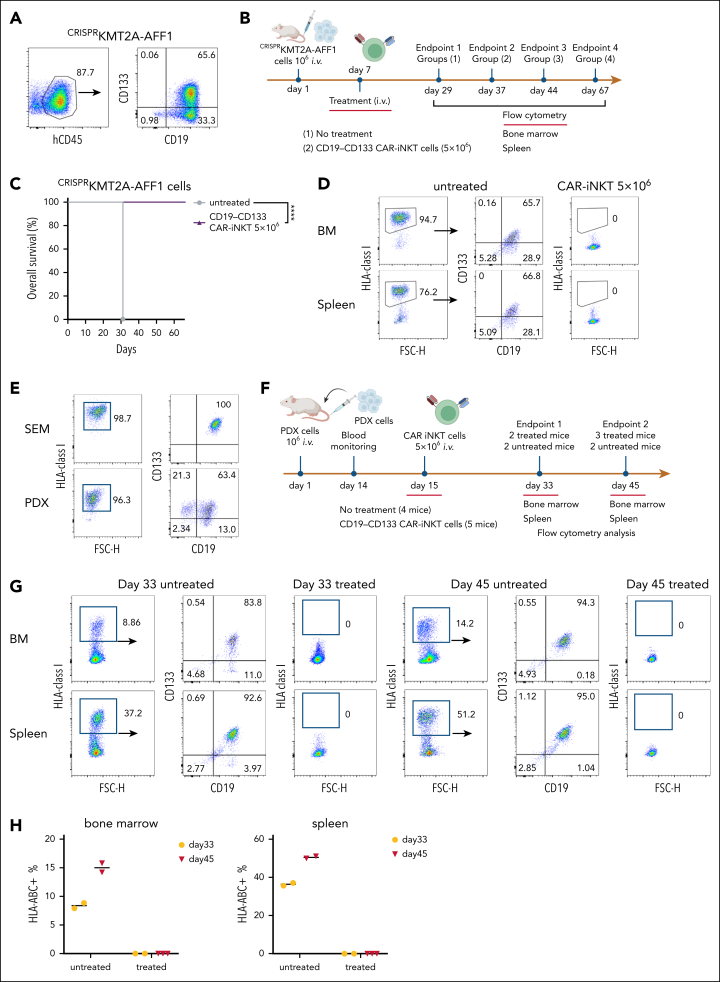
**Bispecific CD19-CD133 CAR-iNKT cells eradicate primary KMT2Ar-ALL with variable CAR target expression.** (A) CD19 and CD133 coexpression pattern in ^CRISPR^KMT2A-AFF1 cells. (B) Schematic of experiment shown in panels C and D. (C) Overall survival of ^CRISPR^KMT2A-AFF1 leukemia-bearing mice treated with 5 × 10^6^ bispecific CAR-iNKT cells (n = 7 mice per group). ∗∗∗∗*P* < .0001. (D) Representative flow cytometric analysis of ^CRISPR^KMT2A-AFF1 leukemia cells as identified with HLA-class I staining in bone marrow and spleen of leukemia-bearing mice treated with 5 × 10^6^ bispecific CAR-iNKT cells. (E) CD19 and CD133 coexpression pattern in PDX KMT2A-AFF1 cells compared with that of SEM cells. (F) Schematic of experiment shown in panels G and H. (G) Flow cytometric analysis of leukemia burden in bone marrow and spleen of untreated and 5 × 10^6^ bispecific CAR-iNKT–treated PDX KMT2A-AFF1 leukemia-bearing mice 18 and 30 days posttreatment. HLA class I^+^ cells were gated on 7-aminoactinomycin D (7-AAD)–negative live cells. (H) Cumulative data of mice treated as shown in panel G (n = 4 control mice, 5 treated mice).

To test the efficacy of the bispecific CAR-iNKT cells against primary blast cells, we used ^CRISPR^KMT2A-AFF1 ALL cells (supplemental Figure 1A,C), in which CD133 was expressed by ∼65% of the CD19^+^ blasts (Figure 3A).

Treatment of ^CRISPR^KMT2A-AFF1 leukemia-engrafted mice (assessed by the presence of >1% leukemic cells in peripheral blood on day 6) with 5 × 10^6^ bispecific CAR-iNKT cells resulted in 100% of ^CRISPR^KMT2A-AFF1 leukemia-bearing mice surviving >60 days (Figure 3B-C) with no detectable HLA-class–positive cells in the BM or spleen, suggesting leukemia elimination (Figure 3D).

Similarly, in a KMT2A::AFF1 patient-derived xenograft model in which leukemia cells express lower levels of CD19 and CD133 than SEM cells (Figure 3E), 5 × 10^6^ bispecific CAR-iNKT cells at day 15 eradicated leukemia in vivo in patient-derived xenograft–engrafted animals (Figure 3F-H). On day 33 and day 45, no human cells were detected in treated animals, as opposed to increasing leukemic burden in untreated mice (Figure 3G-H).

We conclude that bispecific CAR-iNKT cells, when delivered in high doses, effectively eliminate primary KMT2Ar-ALL.

### CAR-iNKT cells are more potent than CAR T cells

**Figure 4.**
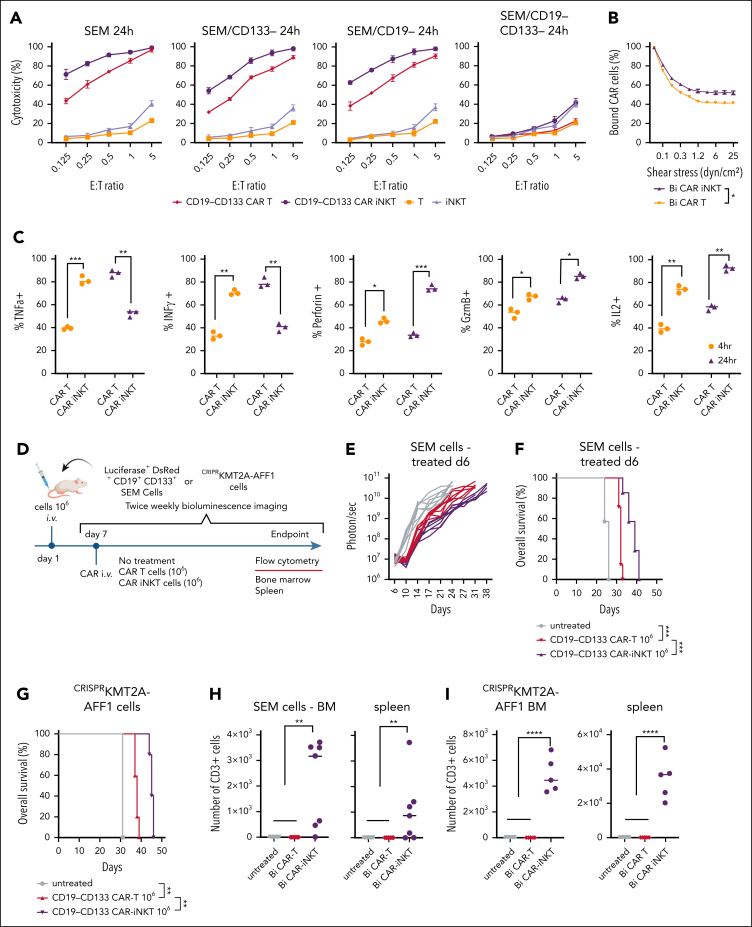

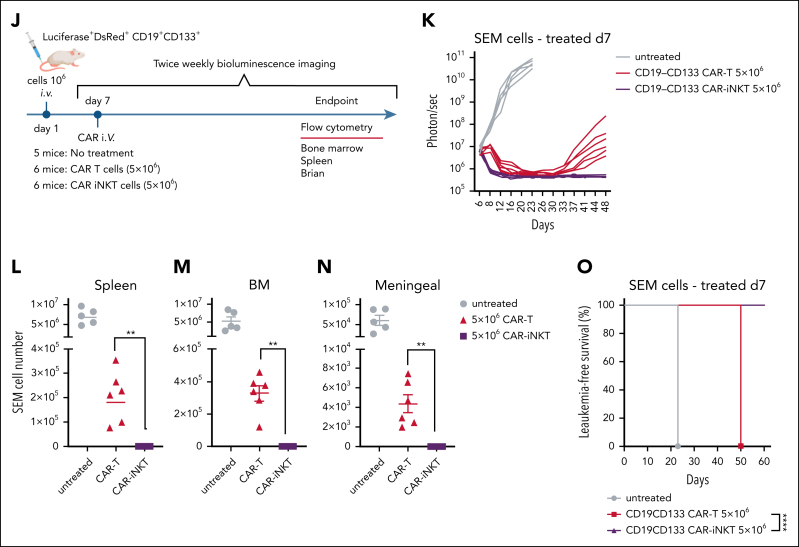
**CAR-iNKT cells are more potent than CAR T cells.** (A) Cytotoxic activity at 24 hours of untransduced T cells and iNKT cells, and of their bispecific CAR-transduced counterparts against parental SEM cells and their single or double CD19/CD133 gene-edited sublines. (B) Avidity assay comparing bispecific CAR T cells vs CAR-iNKT cells against SEM cells. Data are presented as the mean ± SEM of n = 3 experiments. ∗*P* < .05, by 1-way ANOVA. (C) Expression level of indicated cytokines by bispecific CAR T cells and CAR-iNKT cells after 4 and 24 hours coculture with SEM leukemia cells; 1-way ANOVA; ∗*P* < .05; ∗∗*P* < .01; ∗∗∗*P* < .001. (D) Schematic of experiment for panels E-G and H-I. (E-F) Leukemia burden by BLI and overall survival of SEM leukemia-bearing mice treated with indicated dose of bispecific CAR T cells and CAR-iNKT cells (n = 7 mice per group). Log-rank test, ∗∗∗*P* < .001. (G) Overall survival of ^CRISPR^KMT2A-AFF1 leukemia-bearing mice treated with indicated dose of bispecific CAR T cells and CAR-iNKT cells (n = 7 mice per group). Log-rank test, ∗∗*P* < .01. (H) Absolute numbers of CAR T cells and CAR-iNKT cells in BM and spleen at euthanasia of mice treated as shown in panels E-F. One-way ANOVA; ∗∗*P* < .01. (I) Absolute numbers of CAR T cells and CAR-iNKT cells in BM and spleen at euthanasia of mice treated as shown in panel D, 1-way ANOVA; ∗∗∗∗*P* < .0001. (J-K) Schematic of the experiment (J) and leukemia burden assessed by BLI of SEM leukemia-bearing mice treated with 5 × 10^6^ CAR-iNKT cells or CAR T cells (K). (L-N) Absolute numbers of leukemia cells in spleen (L), BM (M), and meningeal space (N) at euthanasia of mice treated as shown in panel K; ∗∗*P* < .01. (O) Leukemia-free survival of mice treated with 5 × 10^6^ CAR-iNKT cells or CAR T cells. Log-rank test, ∗∗∗∗*P* < .0001. E:T, effector to target.

We compared the antileukemic activity of CD19-CD133 CAR-iNKT cells with same donor CAR T-cell counterparts manufactured for 28 and 14 days, respectively (supplemental Figure 4A). CAR-iNKT cells were more cytotoxic than CAR T cells against the parental and gene-edited SEM cells and the RS4;11 cells (Figure 4A; supplemental Figure 4B), and showed higher avidity (Figure 4B). As for SEM cells, bispecific CAR-iNKT cells were also more cytotoxic against ^CRISPR^KMT2A-AFF1 ALL blasts than CAR T cells in vitro (supplemental Figure 4C), and similarly monospecific CD19 and CD133 CAR-iNKT cells were more cytotoxic against parental CD19^+^CD133^+^ SEM and RS4;11 cells and the CD19^+^CD133^–^ KOPN8 KMT2Ar cells when compared with CAR T-cell counterparts (supplemental Figure 4C).

Consistent with their “innate” functional profile, CAR-iNKT cells, upon coculture with SEM cells for 4 and 24 hours, produced similar peak levels of interferon gamma (IFN-γ) and tumor necrosis factor α as CAR T cells, but at an earlier time point, and demonstrated a higher cytotoxic potential as indicated by production of higher amounts of perforin (PRF), granzyme B (GRZB), and interleukin-2 compared with CAR T cells (Figure 4C; supplemental Figure 4D).

In vivo, at the subtherapeutic dose of 10^6^ CAR^+^ cells, CAR-iNKT cell–treated SEM- and ^CRISPR^KMT2A-AFF1 leukemia-bearing mice had a lower disease burden and significantly longer survival than CAR T-cell–treated mice (Figure 4D-G). In both models, while CAR T cells were not detected in the BM and spleen of treated animals at end point, CAR-iNKT cells were readily detectable 1 week later (Figure 4H-I).

Finally, at the therapeutic dose of 5 × 10^6^ CAR^+^ cells, in CAR-iNKT cell–treated SEM leukemia-bearing mice, reduction in leukemia burden was swift and reached undetectable levels within 72 hours after treatment, and remained at significantly lower levels than in CAR T-cell–treated mice throughout the course of the experiment; by contrast, the BLI signal clearly increased in CAR T-cell–treated animals before termination, indicating relapsing disease (Figure 4J-K; supplemental Figure 4E). Assessment of leukemia by flow cytometry in the BM, spleen, and meningeal space upon termination confirmed eradication of leukemia in CAR-iNKT cell–, but not CAR T-cell–treated mice (Figure 4L-N), with the former showing a 100% leukemia-free survival (Figure 4O).

No signs of aGVHD were observed in animals treated with either therapy.

Therefore, CAR-iNKT cells are more potent than CAR T cells at both subtherapeutic and therapeutic doses.

### NKG2D upregulation in CAR-iNKT cells accounts for their ability to outperform CAR T cells in vitro

The leukemia cells we tested had negligible expression of CD1d (supplemental Figures 1A, 4F, and 5A). Consistent with this, cytotoxic activity of bispecific CAR-iNKT cells against SEM cells did not change in the presence of blocking anti-CD1d antibody (supplemental Figure 5A). Therefore, we hypothesized that the mechanism that dictates higher antileukemia activity of CAR-iNKT cells than CAR T cells was CD1d independent, and could involve differential expression of activating NK receptors such as NKG2D, which is known to be expressed on CD4^–^ iNKT cells.^35^^,^^36^

**Figure 5.**
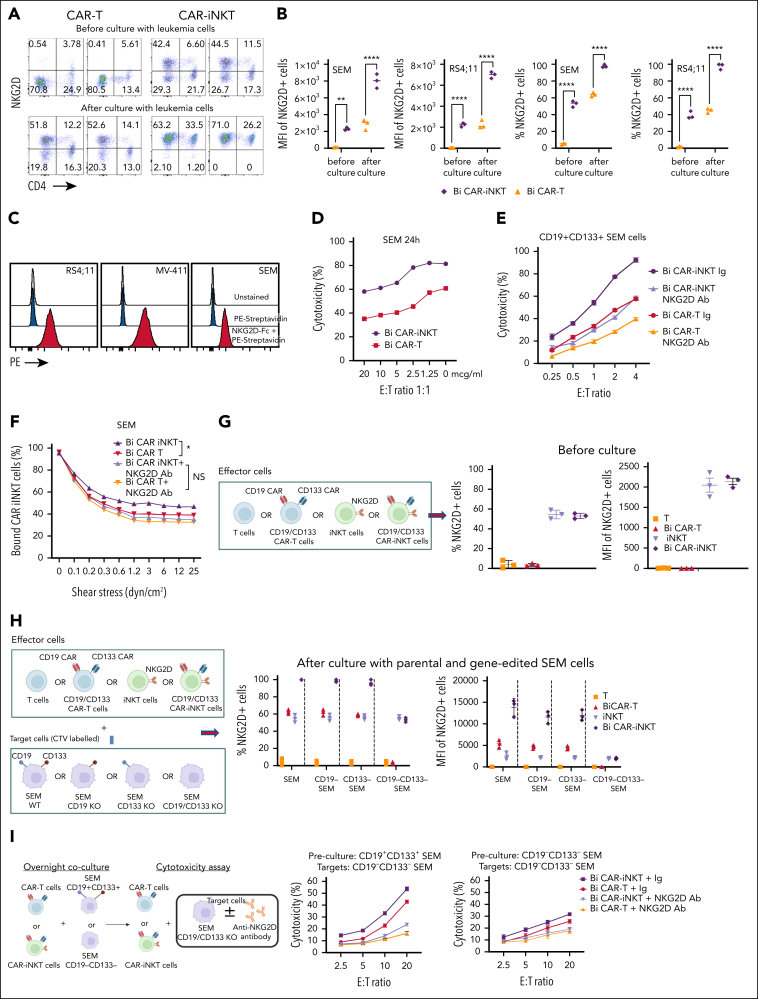
**NKG2D upregulation in CAR-iNKT cells accounts for their ability to outperform CAR T cells in vitro.** (A) Flow cytometric analysis of NKG2D expression on bispecific CD19-CD133 CAR T cells and CAR-iNKT cells before and after overnight coculture with SEM cells. (B) NKG2D expression as measured by per cent of cells and mean fluorescence intensity (MFI) in SEM and RS4;11 KMT2Ar cells. ∗∗*P* < .01, ∗∗∗∗*P* < .0001, by 1-way ANOVA. (C) NKG2D ligand expression in the indicated KMT2Ar leukemia cell lines as assessed by staining with biotinylated NKG2D-Fc protein followed by fluorescent streptavidin. (D-E) Cytotoxicity of bispecific CAR T cells and CAR-iNKT cells that had been precultured with SEM cells against SEM cells in the presence of different concentrations (D), or 5 mg of NKG2D mAb or immunoglobulin (Ig) isotype control (E). (F) Avidity measurement of bispecific CAR-iNKT cells vs CAR T cells against SEM cells in the presence of NKG2D mAb or Ig isotype control. Data are presented as the mean ± SEM. NS, not significant; ∗*P* < .05, by 1-way ANOVA. (G) Left: schematic of experiment. Right: NKG2D expression (% and MFI) in untransduced T cells/iNKT cells and CAR T cells/CAR-iNKT cells before coculture with leukemia cells. (H) Left: schematic of experiment. Right: NKG2D expression (% and MFI) in untransduced T cells/iNKT cells and CAR T cells/CAR-iNKT cells after coculture with the different SEM cell lines as shown. (I) Left: schematic of experiment. Middle: cytotoxicity of bispecific CAR T cells and CAR-iNKT cells that had been precultured with CD19^+^CD133^+^ SEM cells against CD19^–^CD133^–^ SEM cells in the presence of NKG2D mAb or Ig isotype control. Right: cytotoxicity of bispecific CAR T cells and CAR-iNKT cells that had been precultured with CD19^–^CD133^–^ SEM cells against CD19^–^CD133^–^ SEM cells in the presence of NKG2D mAb or Ig isotype control. (A-I) Data are from 2 independent experiments and 2 different iNKT cell donors.

As expected, we found that expression of NKG2D at baseline was significantly higher in bispecific CAR-iNKT cells than CAR T-cell counterparts (Figure 5A-B). Following a 16-hour exposure to SEM or RS4;11 cells, NKG2D expression increased to nearly 100% in CAR-iNKT cells, compared with only a modest increase in CAR T cells (Figure 5A-B). Of note, while only CD4^–^ and not CD4^+^ iNKT cells expressed NKG2D at baseline, upon exposure to leukemia cells, both fractions displayed expression of NKG2D to nearly 100%.

To investigate the functional significance of these observations, we tested the cytotoxic activity of bispecific CAR-iNKT cells and CAR T cells in the presence of an NKG2D blocking mAb or immunoglobulin control. We first confirmed expression of NKG2D ligands on KMT2Ar cell lines by staining with a soluble NKG2D-Fc protein and RNA expression (Figure 5C; supplemental Figure 5A). We found a concentration-dependent decrease in the cytotoxic activity of CAR-iNKT cells and T cells against SEM cells in the presence of NKG2D mAb (Figure 5D-E), suggesting that the higher antileukemic activity of CAR-iNKT cells is largely NKG2D-mediated. Consistent with these results, the higher avidity displayed by bispecific CAR-iNKT cells when compared with CAR T-cell counterparts was also mitigated upon NKG2D blockade (Figure 5F). Using NKG2D-Fc soluble protein to block the NKG2D ligands on target cells also abrogated the enhanced cytotoxicity of CAR-iNKT cells vs CAR T cells, further confirming the role of NKG2D in the differential reactivity of CAR-iNKT cells vs CAR T cells against leukemia (supplemental Figure 5B). Of note, monospecific CAR-iNKT cells displayed the same phenomenon of higher NKG2D-dependent cytotoxicity as compared with their CAR T-cell counterparts (supplemental Figure 5C).

To investigate the mechanism of upregulation of NKG2D expression, we cocultured untransduced or CAR-transduced iNKT cells and T cells with SEM cells expressing CD19 only, CD133 only, both, or none (Figure 5G-H). We found that expression of NKG2D in untransduced iNKT cells and T cells did not increase after exposure to CAR antigen–expressing or nonexpressing SEM cells. By contrast, the baseline expression of NKG2D on CAR-iNKT cells and to a lesser degree on CAR T cells increased after their coculture with CAR target–expressing cells, that is, CD19^+^CD133^+^, CD19^+^CD133^−^ or CD19^−^CD133^+^ SEM cells; however, upon coculture with CD19^−^CD133^−^ SEM cells, there was a failure to upregulate NKG2D (Figure 5H; supplemental Figure 5D). Together these results show that upregulation of NKG2D requires both expression of CARs and their engagement by their corresponding targets on leukemia cells.

We next tested whether CAR-iNKT cells could kill leukemia cells lacking expression of both CAR targets, and the role of NKG2D in this process (Figure 5I). CAR-iNKT cells and to a lesser degree CAR T cells that had been preexposed to CAR target–expressing leukemia cells displayed higher cytotoxicity against CAR target–negative SEM cells than CAR-iNKT cells that had been preexposed to leukemia cells lacking expression of the CAR targets (Figure 5I), and this effect was attenuated by NKG2D blockade. Similarly, CAR-iNKT cells precultured with CD19^+^CD133^+ CRISPR^KMT2A-AFF1 ALL blasts upregulated NKG2D (supplemental Figure 5E), and subsequently were able to kill CAR target–negative SEM cells in an NKG2D-dependent manner (supplemental Figure 5F).

These findings highlight the ability of CAR-iNKT cells to enhance their innate, NKG2D-dependent effector functions in a CAR-dependent and CAR antigen–dependent manner. Such a mechanism explains their higher efficacy compared with T cells, and could help limit immune escape of CAR target–low/negative leukemia cells.

### Ex vivo functional characterization of CAR-iNKT cells at the single cell level

**Figure 6.**
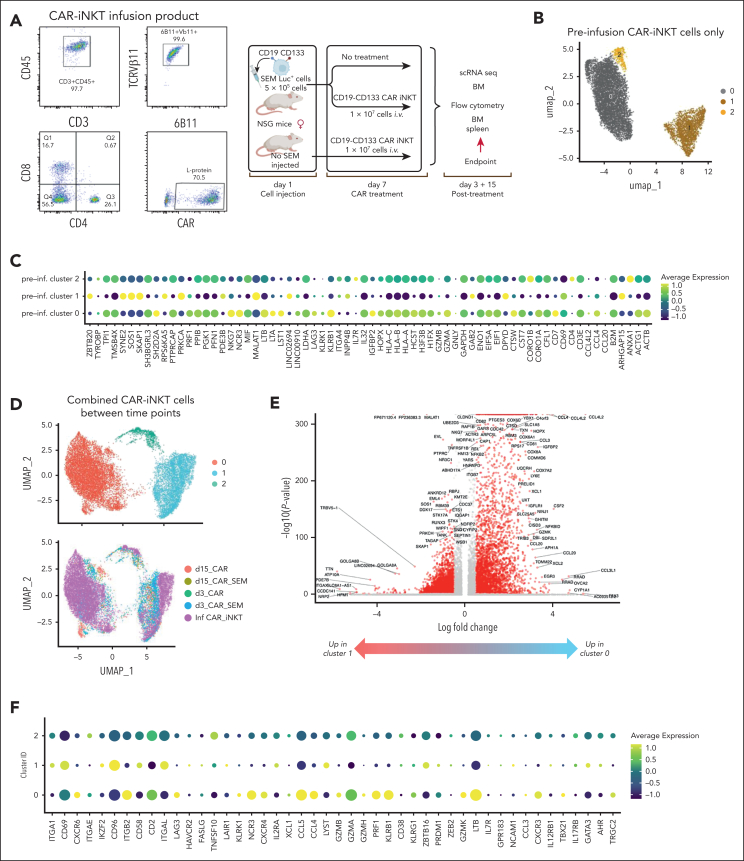

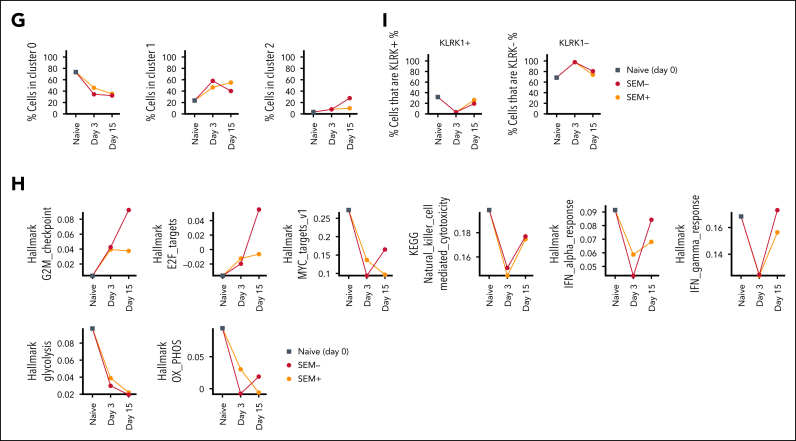
**Ex vivo transcriptome analysis of bispecific CAR-iNKT cells.** (A) Immunophenotypic profiling of preinfusion CAR-iNKT cells and design of the experiment. (B) UMAP embedding of gene expression data of the preinfusion CAR-iNKT cells, and (C) bubble plot showing comparative expression levels of genes of immunological relevance in clusters 0, 1, and 2 from panel B. (D) UMAP embedding of gene expression data of all experimental groups after batch correction colored by cluster ID (top) and by sample (bottom). (E) Volcano plot showing differential gene expression between clusters 0 and 1 (log_2_FC > 1 and *P*adj < .05). (F) Bubble plot showing comparative expression levels of genes of immunological relevance in clusters 0, 1, and 2. (G) Frequency of cells in different clusters over the time course of the experiment. (H) Dynamic variation in the score of indicated enriched pathways between the preinfusion product, and days 3 and 15 CAR-iNKT cells. All pathways are significantly different between samples (*P* < .001). (I) Dynamic variation in the proportion of KLRK1+/− cells between the preinfusion product, and days 3 and 15 CAR-iNKT cells. UMAP, Uniform Manifold Approximation and Projection.

To gain insights into how the functional state of CAR-iNKT cells is altered in vivo upon their engagement with leukemia, we subjected preinfusion CAR-iNKT cells and CAR-iNKT cells isolated at 2 time points (days 3 and 15 after CAR-iNKT cell treatment) from the BM of CAR-iNKT cell–treated, leukemia-bearing or leukemia-free mice (ie, 5 groups) to sc multiomic TCRαβ and transcriptome analysis (Figure 6A). On day 3, no leukemia cells were detected in the BM and spleen of treated animals, and on day 15 CAR-iNKT cells were still readily detectable (supplemental Figure 6A).

First, sc gene expression–based dimensionality reduction of the preinfused CAR-iNKT cells identified 3 clusters (C0-2) with, as expected, the minority of cells expressing CD4 and colocalizing mostly in cluster 2 (Figure 6B). Differential gene expression between CD4^+^ and CD4^−^ CAR-iNKT cells (supplemental Table 1) identified the latter as enriched in a cytotoxic transcriptional signature (*PRF1*, *NKG7*, *NCR3*, *LTA*, *LTB*, *KLRK1* [NKG2D], *KLRB1* [CD161], *GZMB/A*), consistent with their known biology (Figure 6C). sc Gene expression– and TCR expression–based dimensionality reduction of all CAR-iNKT cells identified 3 clusters of cells (C0, C1, and C2) with all 5 groups represented in each cluster (Figure 6D). As expected, although not captured in all cells, the invariant TCRAV10-TRAJ18 sequence was by far the most dominant TRA clonotype (supplemental Figure 6B-D).

Genes overexpressed in cluster 0 vs 1 (n = 1642, Log_2_FC > 1, *P*adj < .05; Figure 6E; supplemental Table 2) were enriched for oxidative phosphorylation and enhanced protein synthetic capacity (Myc Targets V1; MTORC1 signaling; supplemental Figure 6E), suggesting that favorable energy production and protein anabolic pathways support CAR-iNKT cell function. Cluster 0, compared with clusters 1 and 2, emerged with a robust profile of cytotoxic potential with perforin (*PRF1*), granzymes (GZMB, GZMH, GZMK), and activating NK cell receptor (*KLRK1/NKG2D*, *NCR3*, *LTB*) genes preferentially and highly expressed (Figure 6F). Of note, with the exception of *GZMH* and *GZMK*, cells retained their expression of *GZMA*, *GZMB*, and *PRF1* after 3 and 15 days (supplemental Figure 6F).

Differential gene expression analysis between the day 3 groups revealed only a handful of variable genes, whereas a larger number of variable genes were identified between day 15 groups (supplemental Figure 6G; supplemental Table 2). Day 15 iNKT cells from leukemia-bearing mice overexpressed 264 genes, compared with iNKT cells from leukemia-free mice, with the most enriched pathways corresponding to cell cycle (G2M checkpoint, E2F targets; supplemental Figure 6E). Applying a transcriptional score for the cell cycle S phase in CAR-iNKT cells from day 15 leukemia-bearing vs leukemia-free mice showed the former to have a higher proliferative status (*P* < 6.2 × 10^–6^; supplemental Figure 6H). In line with this, while the frequency of C0 declined, the frequency of cells in cluster 2, which is the most proliferative of the 3 clusters (supplemental Figure 6I), increased on day 15 only in leukemia-bearing but not leukemia-free mice (Figure 6G). These data highlight the fact that >10 days after clearing leukemia, CAR-iNKT cells retain robust proliferative potential.

To obtain a dynamic overview of the function of CAR-iNKT cells, we scored pathways enriched between the 5 experimental groups (*P* < 10^–10^), and plotted them against the preinfusion, days 3 and 15 (Figure 6H). We found that proliferation increases in vivo with time, with a higher score in CAR-iNKT cells from leukemia-bearing mice (G2M checkpoint, E2F targets). While on day 3, cytotoxic and IFN-α and IFN-γ responses are blunted, on day 15 iNKT cells they are both restored, with IFN-α and IFN-γ responses scoring higher in CAR-iNKT cells from leukemia-bearing mice. This is mirrored by a reduction in metabolic pathways including glycolysis, but relative preservation of oxidative phosphorylation in day 15 iNKT cells from leukemia-bearing mice. Finally, scoring the frequency of KLRK1/NKG2D-expressing cells shows that after their decline on day 3 they recover on day 15 (Figure 6I).

Together these findings suggest that CAR-iNKT cells retain and enhance their effector and proliferative functions after their in vivo targeting and eradication of leukemia. To corroborate this prediction, we isolated iNKT cells from the BM of bispecific CAR-iNKT cell–treated animals, 5 weeks after treatment (supplemental Figure 7A), and investigated their functional properties in vitro. Explanted CAR-iNKT cells proliferated robustly in the presence of aGalCer (>100-fold over 2 weeks; supplemental Figure 7B), retained the same level of CAR expression as the originally injected cells (supplemental Figure 7C), and following expansion they retained their ability to exert a powerful cytotoxic effect when cultured with CD19^hi^CD133^hi^ or CD19^lo^CD133^lo^ SEM leukemia cells (supplemental Figure 7D). Therefore, even after 4 weeks of preinfusion in vitro expansion, ∼5 weeks of in vivo persistence in leukemia-bearing mice, and another 3 weeks of ex vivo expansion, bispecific CAR-iNKT cells retain robust proliferative and cytolytic antileukemia activity.

### Impact of bispecific CAR-iNKT cells on normal human hematopoiesis

**Figure 7.**
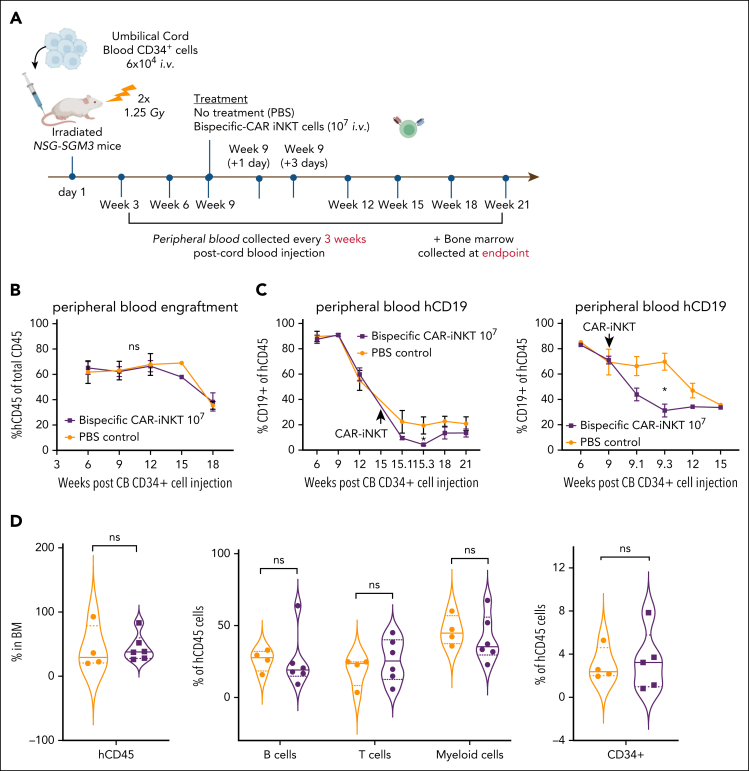
**Impact of bispecific CAR-iNKT cells on hematopoiesis in humanized mice.** (A) Schematic of xenograft experiment to test hematologic toxicity of CD19-CD133 CAR-iNKT cells in vivo. (B) Peripheral blood human CD45 engraftment levels in mice treated with phosphate-buffered saline (PBS; n = 5) or 10^7^ CD19-CD133 CAR-iNKT cells (n = 7). (C) Peripheral blood human CD19^+^ cells in mice treated with PBS (n = 5) or 10^7^ CD19-CD133 CAR-iNKT cells (n = 7), in 2 separate experiments, showing a transient drop in B-cell proportions 1 to 3 days after CAR injection. Unpaired *t* test; ∗*P* < .05. (D) Long-term engraftment in the BM of mice treated with PBS or 10^7^ CD19-CD133 CAR-iNKT cells. From left to right: proportion of hCD45 cells in BM (PBS, n = 4, CAR, n = 6); B cells, T cells, and myeloid cells in the BM expressed as proportion of hCD45^+^ cells (PBS, n = 4, CAR, n = 6); and immature CD34^+^ cells in the BM expressed as proportion of hCD45^+^ cells (PBS, n = 4, CAR, n = 5). Median denoted by solid lines, and dashed lines denote quartile distribution within the sample set. ns, not significant.

Given the expression of CD133 by a proportion of normal primitive HSPCs (supplemental Figure 1D), we used a humanized xenograft model to assess potential off-tumor effects of CD19-CD133 bispecific CAR-iNKT cells by transplanting cord blood CD34^+^ cells into sublethally irradiated NSG-SGM3 mice (Figure 7A). In 2 independent experiments, engrafted mice (confirmed by hCD45 expression in >1% of PB cells; Figure 7B and supplemental Figure 8A) were treated with either phosphate-buffered saline or 10^7^ bispecific CAR-iNKT cells. On-target, off-leukemia activity of CAR iNKT cells was confirmed by a significant reduction in the frequency of CD19^+^ B cells in the PB at day 1 and day 3 after CAR-iNKT cell injection, compared with controls (Figure 7C; supplemental Figure 8B). Long-term engraftment in PB (18 weeks) or BM (at cull 18-21 weeks after transplant) showed no significant difference between CAR-iNKT cell–treated and phosphate-buffered saline–treated mice either in total hCD45^+^ cells or, in BM, in the frequencies of B, T, myeloid or CD34^+^ cells within the hCD45 compartment (Figure 7D; supplemental Figure 8C). This suggests there is no significant CAR-iNKT cell–mediated toxicity against primitive HSPCs.

## Discussion

A combination of chemoresistance and a high risk of central nervous system disease contribute to the adverse prognosis of KMT2Ar-ALL, including infant ALL. Here we deploy allogeneic iNKT cells, equipped with bispecific CD19-CD133–targeting CARs that engage single or coexpressed leukemia-associated antigens in this disease.

Enhanced avidity of bispecific CAR-iNKT cells, in line with recent work,^19^^,^^37^^,^^38^ correlated with in vivo efficacy. This would be particularly important for the effective targeting of CAR antigen–low disease, and would potentially mitigate subsequent CAR target–low relapse often seen in KMT2Ar-ALL and other forms of ALL treated with CD19 CAR T cells.^8^^,^^11^

Our study clearly showed the curative potential of CAR-iNKT cells when used at higher dose levels in vivo. This is in line with recent preclinical data showing that failure of first-line immunotherapy is often not due to tumor-intrinsic mechanisms of immune escape, but simply due to insufficient numbers of effector cells reaching all tumor sites when relatively low therapeutic doses are used.^39^ These findings should inform clinical development, and call for use of high doses of CAR-iNKT cells in future clinical trials.

A major advantage of bispecific CAR-iNKT cells is their impressive ability to confer protection from and eradicate established meningeal leukemia, which affects a higher proportion of infants with KMT2Ar-ALL.^40^^,^^41^ We previously reported CAR-iNKT cell–mediated clearance in brain lymphoma,^26^ mediated by higher expression of VLA-4^26^ (an integrin required for endothelial adhesion and crossing of the blood-brain and choroid plexus–brain barriers) than CAR T cells.^26^

Previous work demonstrated the importance of NKG2D expression by CD4^–^ iNKT cells, and by CAR-iNKT cells for enhancing their activation independent of the invariant TCR-CD1d interaction when engaged by NKG2D stress ligands.^35^^,^^42^ Here, we demonstrate that a novel process of dynamic NKG2D expression by both CD4^–^ and CD4^+^ iNKT cell subsets allows targeting of leukemia cells with variable expression of CAR antigens more efficiently than by CAR T cells, in an NKG2D-dependent manner. These findings are analogous to TCR–major histocompatibility complex class I dependent upregulation of NKG2D in conventional CD8^+^ T cells, allowing them to target tumor cells with loss of major histocompatibility complex molecules in an NKG2D-dependent manner.^43^

Importantly, our study showed that at dose levels that induce B-cell depletion, bispecific CD19-CD133 CAR-iNKT cells did not significantly affect long-term hematopoiesis, in line with a clinical trial of autologous CD133 CAR T cells in patients with solid tumors that reported only transient grade 2 hematologic toxicity.^44^

We propose that given the logistic difficulties of autologous T-cell collections in infants, future clinical application would likely employ allogeneically sourced, bispecific CAR-iNKT cells as a swift bridging therapy to induce remissions in relapsed/refractory KMT2Ar-ALL prior to allo-hematopoietic stem cell transplantation.^45^ Of note, a bispecific CAR-iNKT cell approach can encompass other KMTA2r-ALL targets such as FLT3.^46^ After extensive preclinical development,^42^^,^^47^, ^48^, ^49^ allogeneic CAR-iNKT cells and iNKT cell–based immunotherapeutics are at the verge of clinical development, with early clinical results suggesting high efficacy with, as predicted, lack of aGVHD and, importantly, mild or no cytokine release syndrome and neurotoxicity.^50^^,^^51^ Of note, due to the limitations of the NSG model, our work does not address previously reported indirect antitumor effects of iNKT cell–based immunotherapy, including depletion of CD1d-expressing immunosuppressive, tumor-associated myeloid cells, and activation of antitumor T- and NK-cell responses.^30^^,^^47^^,^^52^, ^53^, ^54^ Finally, we did not address whether antirejection strategies would further enhance the antileukemia efficacy of CAR-iNKT cell immunotherapy.

We conclude that preclinical bispecific CAR-iNKT cell immunotherapy is a very effective treatment for aggressive KMT2Ar-ALL. It outperforms CAR T-cell immunotherapy in an NKG2D-dependent manner, eradicates leptomeningeal leukemia, and has the potential to protect from immune escape with CAR target antigen–low disease without discernible hematologic toxicity. These findings provide the basis for clinical development of bispecific CD19-CD133 CAR-iNKT cells as an off-the-shelf treatment for KMT2Ar-ALL.

Conflict-of-interest disclosure: A.K., T.A.M., A.R., H.R., N.E., B.L., C.H., and R.B.-R. are coauthors of a patent based on this work. A.K. chairs the scientific advisory board of and holds share options in Arovella Therapeutics. T.A.M. is a shareholder in and consultant for Dark Blue Therapeutics. R.B.-R. is a cofounder of Theraimmune and Alchemab Therapeutics Ltd, and a consultant for Alchemab Therapeutics. The remaining authors declare no competing financial interests.

## Supplementary Material

Supplemental Methods, References, Figures, and Table 3
