# Supplementary material for: Off-the-shelf dual CAR-iNKT cell immunotherapy eradicates medullary and leptomeningeal high-risk KMT2A-rearranged leukemia
Source: Blood. 2025 Sep 4;147(2):180–96. doi: 10.1182/blood.2025029302 (PMC12824662; doi:10.1182/blood.2025029302)
Supplement: Supplemental Methods, References, Figures, and Table 3 [file BLOOD_BLD-2025-029302-mmc1.pdf]

**SUPPLEMENTAL INFORMATION**

# Bi-specific CAR-iNKT cell immunotherapy for high-risk KMT2A-rearranged leukemia outperforms CAR-T in an NKG2D-dependent manner and eradicates leptomeningeal disease

Hongwei Ren[1]*, Natalina Elliott[2]*, Bryan Lye[1], Mohammad Umer Sharif Shohan[3], Joe W Cross[2], Lucy Field[2], Kanagaraju Ponnusamy[1], Siobhan Rice[4], Thomas Jackson[2], Ilia Leontari[1], Nouhad El Ouazzani[5], Rebecca Thomas[5], Sarah Inglott[2,5], Jack Bartram[5], Owen Smith[6,7,8], Jonathan Bond[7,8], Irene AG Roberts[2,4], Christina Halsey[9], Rachael Bashford-Rogers[3,10], Thomas A Milne[4], Anindita Roy[2,4,5], Anastasios Karadimitris[1,11]

[1]Centre for Haematology, Department of Immunology and Inflammation, Imperial College London, London, UK
[2]Department of Paediatrics, University of Oxford, Oxford, UK
[3]Department of Biochemistry, University of Oxford, Oxford, UK
[4]MRC Molecular Haematology Unit, MRC Weatherall Institute of Molecular Medicine, University of Oxford, Oxford, UK
[5]Department of Haematology, Great Ormond Street Hospital, London, United Kingdom
[6]Trinity College, University of Dublin, Dublin, Ireland
[7]Systems Biology Ireland, School of Medicine, University College Dublin, Ireland
[8]Children's Health Ireland at Crumlin, Dublin, Ireland
[9] Wolfson Wohl Cancer Research Centre, School of Cancer Sciences, College of Medical Veterinary and Life Sciences, University of Glasgow, Glasgow, UK
[10]Cancer Research UK Oxford Centre, University of Oxford, Oxford, UK
[11]Department of Haematology, Imperial College Health NHS Trust, Hammersmith Hospital, London, UK
*equal contribution

**Correspondence**
Anastasios Karadimitris: a.karadimitris@imperial.ac.uk
Anindita Roy: anindita.roy@paediatrics.ox.ac.uk
Thomas Milne: thomas.milne@imm.ox.ac.uk

## Supplementary Methods

### Primary human samples

*iNKT and T cells*: Peripheral blood mononuclear cells (PBMCs) obtained from healthy donors were isolated by density gradient centrifugation and used as a source of CD3+ lymphoid cells for CAR engineering.

*Human fetal hematopoietic stem and progenitor cells*: These were provided for the purposes of this research by the Human Developmental Biology Resource (HDBR, www.hdbr.org), regulated by the UK Human Tissue Authority (HTA, www.hta.gov.uk) and covered under ethics granted by NHS Health Research Authorities: North East – Newcastle & North Tyneside Research Ethics Committee (REC: 23/NE/0135) and London - Fulham Research Ethics Committee (23/LO/0312). Informed consent was obtained from all participants, who donated human fetal tissue for research without receiving any monetary compensation. FL samples used for CRISPR/Cas9 KMT2A-AFF1 translocation experiments underwent CD34 magnetic bead selection at the time of sample processing and were cryopreserved for future use as described[1]. Cord blood samples were obtained from NHSBT under ethical approval (REC: 21/LO/0195) and CD34 cells were selected as above.

*Leukemia samples*: ALL patient samples were obtained from VIVO Biobank, UK after appropriate review of our research project to ensure that it was covered under their ethics approval granted by NHS HRA South West - Central Bristol Research Ethics Committee (REC: 23/EM/0130). Two infant KMT2Ar ALL samples were obtained from Our Lady's Children's Hospital, Crumlin, Dublin, Ireland and one from Oxford University Hospital Trust under ethical approval (REC: 21/LO/0195). Informed consent was obtained from all participants or those with parental responsibility, and participants did not receive any monetary compensation. Infant and paediatric KMT2Ar ALL samples from patients being treated at Great Ormond Street Hospital for Children, London were analysed by flow cytometry as part of their diagnostic workup after informed consent. All patient samples/data were anonymized at source, assigned a unique study number and linked.
Patient derived xenografts: KMT2Ar ALL PDX cells were provided by the Halsey lab (Glasgow).

Model cells of infant B- acute lymphoblastic leukemia were derived by introducing a *KMT2A -AFF1* translocation into CD34 selected human fetal liver cells using CRISPR and transplanted into NSG mice for *in vivo* expansion ($^{CRISPR}$KMT2A-AFF1 ALL) as described in [2].

### Cell lines

SEM, RS4;11 and KOPN8 cell lines were available in the Milne lab. SEM cell line was further modified in the following ways: SEM cells were transduced with non-replicative MIGR1 retrovirus to co-express Luciferase and dsRed as an expression marker. All cell lines were

purchased and maintained under recommended conditions. SEM cells were cultured in IMDM (Gibco) supplemented with 10% FCS. RS4;11 and KOPN-8 cells were cultured in RPMI (Gibco) supplemented with 10% FCS.

**CRISPR/Cas9 gene editing of SEM cells**

*CD19* and *PROM1* knockouts were performed in SEM cells using pools of targeted CRISPR RNP guides purchased from Synthego (**Suppl Table 3**) and following the recommended procedures and concentrations for the Neon Transfection System (Invitrogen: MPK1096) and Cas9 Nuclease V3 (IDT: 1081059)[2]. Knockout cells recovered in standard culture conditions for 3-7 days post-transfection before immunophenotyping was performed to select for complete knockout before continuing on to additional experiments.

**CAR constructs**

For the anti-CD19 single-target CAR, we employed the FMC63 antibody clone, fused with the hinge, transmembrane, and partial intracellular domain of CD8α (amino acids 128-210 of CD8α), followed by the CD28 co-stimulatory domain and CD3ζ activation domain. The anti-CD133 single-target CAR was constructed using the AC133 antibody clone, incorporating the same CD8α components as the anti-CD19 CAR, but coupled with the 4-1BB co-stimulatory domain and CD3ζ activation domain. The bi-specific CD19-CD133 dual CAR was engineered by linking the aforementioned single-target CARs via a T2A peptide sequence. The schematic structures of these CAR constructs are illustrated in Figure 1a. Following synthesis of the complete coding sequences, these constructs were cloned into the pSIEW lentiviral transfer plasmid using appropriate restriction enzyme digestion and ligation approaches.

1, CD19 CAR

MALPVTALLLPLALLLHAARPDIQMTQTTSSLSASLGDRVTISCRASQDISKYLNWYQQKPDGTVKLLIYHT
SRLHSGVPSRFSGSGSGTDYSLTISNLEQEDIATYFCQQGNTLPYTFGGGTKLEITKAGGGGSGGGGSEVKL
QESGPGLVAPSQSLSVTCTVSGVSLPDYGVSWIRQPPRKGLEWLGVIWGSETTYYNSALKSRLTIIKDNSKS
QVFLKMNSLQTDDTAIYYCAKHYYYGGSYAMDYWGQGTSVTVSSFVPVFLPAKPTTTPAPRPPTPAPTIAS
QPLSLRPEACRPAAGGAVHTRGLDFACDIYIWAPLAGTCGVLLLSLVITLYCNHRNRSKRSRLLHSDYMNM
TPRRPGPTRKHYQPYAPPRDFAAYRSRVKFSRSADAPAYQQGQNQLYNELNLGRREEYDVLDKRRGRDPE
MGGKPRRKNPQEGLYNELQKDKMAEAYSEIGMKGERRRGKGHDGLYQGLSTATKDTYDALHMQALPPR

CD8α signal peptideFMC63 light chainGGGGS*2 linkerFMC63 heavy chainCD8α extracellular domain, hinge and transmembrane domainCD28 costimulatory domainCD3ζ stimulation domain

2, CD133 CAR

MALPVTALLLPLALLLHAARPDVVVTQTPLSLPVSFGDQVSISCRSSQSLANSYGNTYLSWYLHKPGQSPQ
LLIYGISNRFSGVPDRFSGSGSGTDFTLKISTIKPEDLGMYYCLQGTHQPYTFGGGTKLEIKGGGGSGGGGS
QVQLQQSGAELVRPGASVKLSCKASGYTFSDFEMHWVKQTPVHGLEWIGDIDPGTGDTAYNLKFKGKAT
LTTDKSSSTAYMELRSLTSEDSAVYYCTLGAFVYWGQGTLVTVSAFVPVFLPAKPTTTPAPRPPTPAPTIASQ

PLSLRPEACRPAAGGAVHTRGLDFACDIYIWAPLAGTCGVLLLSLVITLYCNHRNKRGRKKLLYIFKQPFMRPVQTTQEEDGCSCRFPEEEEGGCELRVKFSRSADAPAYQQGQNQLYNELNLGRREEYDVLDKRRGRDPEMGGKPRRKNPQEGLYNELQKDKMAEAYSEIGMKGERRRGKHDGLYQGLSTATKDTYDALHMQALPPR

CD8α signal peptideAC133 light chainGGGGS*2 linkerAC133 heavy chainCD8α extracellular domain, hinge and transmembrane domain41-BB costimulatory domainCD3ζ stimulation domain

3, Bi-specific CD19-CD133 CAR

MALPVTALLLPLALLLHAARPDIQMTQTTSSLSASLGDRVTISCRASQDISKYLNWYQQKPDGTVKLLIYHTSRLHSGVPSRFSGSGSGTDYSLTISNLEQEDIATYFCQQGNTLPYTFGGGTKLEITKAGGGGSGGGGSEVKLQESGPGLVAPSQSLSVTCTVSGVSLPDYGVSWIRQPPRKGLEWLGVIWGSETTYYNSALKSRLTIIKDNSKSQVFLKMNSLQTDDTAIYYCAKHYYYGGSYAMDYWGQGTSVTVSSFVPVFLPAKPTTTPAPRPPTPAPTIASQPLSLRPEACRPAAGGAVHTRGLDFACDIYIWAPLAGTCGVLLLSLVITLYCNHRNRSKRSRLLHSDYMNMTPRRPGPTRKHYQPYAPPRDFAAYRSRVKFSRSADAPAYQQGQNQLYNELNLGRREEYDVLDKRRGRDPEMGGKPRRKNPQEGLYNELQKDKMAEAYSEIGMKGERRRGKHDGLYQGLSTATKDTYDALHMQALPPRRAEGRGSLLTCGDVEENPGPMALPVTALLLPLALLLHAARPDVVVTQTPLSLPVSFGDQVSISCRSSQSLANSYGNTYLSWYLHKPGQSPQLLIYGISNRFSGVPDRFSGSGSGTDFTLKISTIKPEDLGMYYCLQGTHQPYTFGGGTKLEIKGGGGSGGGGSQVQLQQSGAELVRPGASVKLSCKASGYTFSDFEMHWVKQTPVHGLEWIGDIDPGTGDTAYNLKFKGKATLTTDKSSSTAYMELRSLTSEDSAVYYCTLGAFVYWGQGTLVTVSAFVPVFLPAKPTTTPAPRPPTPAPTIASQPLSLRPEACRPAAGGAVHTRGLDFACDIYIWAPLAGTCGVLLLSLVITLYCNHRNKRGRKKLLYIFKQPFMRPVQTTQEEDGCSCRFPEEEEGGCELRVKFSRSADAPAYQQGQNQLYNELNLGRREEYDVLDKRRGRDPEMGGKPRRKNPQEGLYNELQKDKMAEAYSEIGMKGERRRGKHDGLYQGLSTATKDTYDALHMQALPPR

CD8α signal peptideFMC63 light chain and heavy chainGGGGS*2 linkerAC133 light chain and heavy chainCD8α extracellular domain, hinge and transmembrane domainCD28 costimulatory domainCD3ζ stimulation domain

**CAR-iNKT and CAR-T generation**

TCRVα24Jα18+ lymphocytes were immunomagnetically sorted from PBMC using anti-human iNKT cell microbeads (Miltenyi Biotec). Purified iNKT cells were seeded in 24- or 48-well plates at a 1:1 ratio with irradiated (3500 rad) autologous mononuclear cells (iAPC) and activated with Dynabeads Human T-Activator CD3/CD28 (Gibco™) at a 1:1 beads-to-cell ratio in T cell medium at a density of 1-5 x $10^4$ cells per ml. IL-15 (Miltenyi Biotec) at 30 IU/ml and 150 IU/ml was added at the time of seeding and 12 hours later, respectively. Within 48 hours, activated iNKT cells were transduced with concentrated CAR lentivirus using an MOI of 2-5 in the presence of 8 μg/ml pre-coated retronectin, with spinoculation for 90 minutes at 1000G. After 8-12 hours, cells were resuspended in fresh medium supplemented with 150 IU/ml of IL-15 and allowed to rest for 4 days before assessment of viability and CAR expression. CAR transduction efficiency was determined by flow cytometry as the percentage of L-protein+ cells relative to untransduced controls. CAR+ cells were re-stimulated with a 1:1 ratio of irradiated C1R-CD1d cells loaded with αGalCer (100 ng/ml), IL-15 (30 IU/ml), and with an additional 150 IU/ml of IL-15 added 12-24 hours later.

Subsequently, cells were expanded for 14 to 35 days, assessed for purity, and used for in vitro assays. Alternatively, CAR iNKT cells were harvested during the exponential growth phase, cryopreserved in 10% DMSO, and stored in liquid nitrogen until use. Untransduced iNKT were generated in the same way, with the omission of lentiviral transduction step. CAR-T were generated as previously described[3,4].

### *In vitro* assays

*Flow cytometry***.** Cells were stained with fluorophore-conjugated monoclonal antibodies in PBS with 2% FBS and 1mM EDTA for 30 minutes and analyzed via LSR Fortessa X50 or FACS sorted via BD Aria instruments using BD FACSDiva software (v8.0.2). Antibodies used are detailed in **Suppl Table 4**. Flow cytometry antibodies were validated by titration in-house using primary human fetal mononuclear cells (MNC) or NSG mouse BM. Analysis was performed using FlowJo software (v10.7.1) where gates were set using unstained and fluorescence minus one (FMO) controls.

*Cytotoxicity assays.* These were performed as previously described[3]. Briefly, CellTrace™ Violet (Invitrogen)-labelled targets were incubated at the indicated ratios with effector cells for 4 or 16-24 hours. As controls, targets and effectors alone were simultaneously incubated to determine spontaneous cell death. Cells were then harvested and 7-AAD was added prior to flow cytometric analysis on BD LSR Fortessa Flow Cytometer, using BD FACSDiva software version 6.0. Specific cytotoxic activity was determined as ((% sample (7-AAD+, Violet+) − % spontaneous (7-AAD+, Violet+)) / (100 - %spontaneous (7-AAD+, Violet+)) x 100. All assays were run in duplicates or triplicates and analysed using FlowJo 10.9.0.

Intracellular cytokine expression assays were performed as previously described[3,4].

### Flow-chamber avidity

A µ-VI 0.4-A 6 channel slide (Ibidi) and a pump power system were used for the cell-binding avidity assay. Cell lines or primary leukemia  cells, were attached to poly-L-lysine-coated chips as a monolayer for at least 3 hours prior to testing.  CellTrace™ Far Red -labelled (Invitrogen) target cells were allowed to bind for 5 minutes before the flow rate and pressure were ramped up. The flow rate was increased from 0 to 25.6 ml/min of flow through the chamber, with real-time imaging using a EVOS M5000 Imaging System (Thermo Fisher) conducted under both static and flow conditions. Counting of target cells remaining attached at each flow rate was analysed using ImageJ software.

### *In vivo* experiments

*Animals.* All experiments were performed under two separate project licenses approved by the UK Home Office under the Animal (Scientific Procedures) Act 1986 after approval by the Oxford and Imperial College Animal Welfare and Ethical Review Bodies; and in accordance with the principles of 3Rs (replacement, reduction and refinement) in animal research.

Experimental animals were 6–8-week-old female NOD.Cg-PrkdcscidIl2rgtm1Wjl/SzJ (NSG) mice or 6-week-old female NSGS mice. Mice were housed in IVC cages, and kept at a 12-hour light/dark cycle, 21-22°C temperature and 45-65% relative humidity. They had red tunnels or houses and balconies in the cages as enrichment.

**Bioluminescence Imaging (BLI)**

BLI were collected on an IVIS Lumina XR III Imaging System using Living Image software (PerkinElmer). Mice were anesthetized and maintained under inhalational anaesthesia via a nose cone with 2% isoflurane (Zoetis UK)/medical oxygen. A single intraperitoneal (IP) injection of 150 mg/kg D-luciferin (Goldbio) in PBS was administered to all mice 10 minutes before scanning. Up to three mice were imaged simultaneously in a 12.5 cm field of view (FOV) with a minimum target count of 30,000 and exposure times ranging from 0.5 to 3 minutes at medium binning, with additional images acquired at low binning levels to maximize sensitivity and spatial resolution where required. Both ventral and dorsal scans were acquired for each mouse. The dorsal and ventral signals were quantitated separately through region of interest (ROI) analysis using Living Image software (Aura-4.0.8) and expressed in radiance (units of photons/sec) as a total signal summation normalized to the ROI area. Where required, normalized background signal from similarly sized ROIs was subtracted

**Leukemia Models**

*SEM model.* Six-week-old NOD/SCID/IL-2Rγ-null (NSG) female mice (Charles River, UK) were handled in accordance with the 1986 Animal Scientific Procedures Act and under a United Kingdom Government Home Office–approved project licence PP8553679. The animals were housed at the Hammersmith Central Biomedical Services (CBS) facility, Imperial College London. On day 1, all animals were injected with 5 × 10^6 or 1 x 10^6 luciferase-expressing SEM cells via the tail vein (*iv*), followed by bioluminescence imaging (BLI) monitoring on day 6 to confirm engraftment. On day 7, day 12, or day 16, the mice were randomized to either no treatment or immunotherapy with CAR-T or CAR-iNKT cells generated from the same donor. Thereafter, BLI was performed twice a week until day 21 and weekly until the end of the experiment. All mice were euthanized according to protocol when either experimental or humane endpoints were reached.

$^{CRISPR}$*KMT2A-AF4 ALL model:* As with the SEM cells, on day 1, all animals were injected with 1 × 10^6 $^{CRISPR}$*KMT2A-AF4* cells via the tail vein (iv), followed by tail vein blood collection to determine HLA-ABC or CD19, CD133 expression by flow cytometry on day 6 to confirm engraftment. On day 7, the mice were randomized to either no treatment or immunotherapy with CAR-T or CAR-iNKT cell generated from the same donor. All mice were euthanized according to protocol when either experimental or humane endpoints were reached.

**CAR-iNKT hematologic toxicity assays in humanised mice.** All experiments were carried out under a United Kingdom Government Home Office–approved project licence PP2666723. 7–9-week-old NSGS mice (n=12) were sub-lethally irradiated with two doses of 1.25Gy six hours apart (2.5Gy total) and injected via the tail vein with 60,000 cord blood CD34+ cells. Engraftment was monitored by peripheral blood sampling every 3 weeks. Engrafted mice (>1% human CD45 cells in peripheral blood), were divided into control group (received PBS only) or treatment group, treated with 10 million bi-specific CD19-CD133 CAR-iNKT cells at either 9 weeks or 15 weeks post CB CD34+ transplantation. Additional blood samples were taken at D+1 and D+3 post CAR-iNKT injection. Animals were monitored regularly using a standardized physical scoring system, and any mouse found to be in distress was humanely killed. All well mice were culled between 15-21 weeks post CB transplantation to assess long term bone marrow engraftment. Bone marrow was harvested from these mice for analysis.

**Brain histopathology**

Murine heads were stripped of soft tissues, fixed in 10% neutral-buffered formalin (CellPath) and decalcified in Hilleman and Lee EDTA solution (5.5% EDTA in 10% formalin) for 2–3 weeks. Following paraffin embedding, hematoxylin and eosin staining (Sigma-Aldrich) was performed on 5-mm brain sections. Anti-CD19 immunohistochemistry on paraffin-embedded sections was performed as previously described[5].

Imaging used Axiostar Plus or Axio Imager M2 microscopes with AxioVision and ZEN software (Carl Zeiss, Cambridge, United Kingdom). CNS infiltration was assessed by an experienced pathologist (Halsey) blinded to treatment allocation. 5-6 coronal slices and 2 blocks per head were examined and each block was assigned a score from 0= no infiltrate seen, 1= scattered occasional cells, 2= mild infiltrate, 3=moderate infiltrate, 4= heavy infiltrate.

**Assessment of leptomeningeal leukaemia by flow-cytometry**

This was performed as we previously described[6]. In brief, SEM cells were isolated from the leptomeningeal space and leptomeninges after opening the skull and harvesting the brain. The inner surfaces of the skull were gently scraped and washed with PBS, while the intact brain was washed in PBS, then both suspensions were combined and passed through a 40µm cell strainer (Fisher Scientific). In order to enrich the viable mononuclear cells, the cell suspension was layered on 2:1 Lymphoprep and centrifuged at 800 x g for 30 minutes at 4°C.

**Single cell transcriptome-TCR combined analysis**

*Experimental design.* Bone marrows from either day 3 or day 15 post-CAR-iNKT injection were pooled from 3 NSG mice per group of mice that either did or did not receive SEM cells, before CAR-iNKT enrichment and purification.

*Ex vivo selection of iNKT.* CAR-iNKT cells were enriched by magnetic bead selection following the manufacturers protocol against human CD2 (Miltenyi 130-091-114). CD2 positive cells were then further purified by FACS sorting staining using Labelling Check Reagent (Miltenyi

130-122-219) and 7AAD viability stain (Cayman 11397) with additional antibodies against mouse-CD45, human-CD45, and human CD19. The number of CD2+ cells recovered were 2768 to 7645 per pool (from 3 mice). In addition, 30,000 pre infusion CAR-iNKT cells from the same donor were also FACS sorted for single cell analysis. We then immediately proceeded with the TCR/RNAseq protocol for the sorted cells (10x genomics CG000331 Rev C).

**Single Cell Multi-omics sequencing and pre-processing**

scRNAseq transcriptome processing was performed using the Chromium 10x system involving GEM generation, post GEM-generation clean-up, cDNA amplification and DNA quantification. The library was sequenced using the Illumina NovaSeq platform. Chromium Single Cell Immune Profiling Reagent Kits v1.1 solution was used to deliver a scalable microfluidic platform for gene expression (GEX) and VDJ TCR profiling. Libraries were generated and sequenced from the cDNAs and 10x Barcodes were used to associate individual reads back to the individual partitions.

The analysis pipeline applied to process Chromium single-cell data to align reads and generate feature-barcode matrices was performed as previously described[7]. The raw FASTQ files from scRNAseq and TCR sequencing were processed using Cellranger v7.1.0 in two separate steps. Briefly, gene expression FASTQ files were processed using Cellranger count to perform alignment, filtering, barcode counting, and UMI counting, using 10X Genomics' GRCh38 v2020-A reference. For the TCR V(D)J analysis 'cellranger vdj' command was used taking GRCh38-alts-ensembl-7.1.0 as the reference to align, assemble and annotate T cell receptor sequence.

**Filtering, doublet detection and batch correction**

For each sample, cells with fewer than 500 transcripts or 500 genes of >15% mitochondrial genes were filtered out. Normalisation and scaling were done using the standard Seurat pipeline. Principal component analysis (PCA) was performed on 10,000 highly variable genes (HVGs), excluding highly variable genes encoding for TCR variable chain, ribosomal proteins, heat shock proteins, mitochondrial proteins, cell cycle proteins, HLA, and noise-related genes (MALAT1, JCHAIN, XIST). These were used to compute 50 principal components, then *Harmony* was performed for batch correction[8], UMAP for dimensionality reduction, and the Louvain algorithm was used for clustering (resolution: 0.02).

**TCR-seq analysis**

*scIsoTyper* was used to assign most probable BCR IGH and IGK/L chains per droplet (based on nUMIs) and most probable TCR TRA and TRB chains per droplet (based on nUMIs)[9]. Briefly, *scIsoTyper* performs the following steps: (1) batches TCR data for IMGT submission; (2) parses the IMGT annotation results, for the quantification of V/J gene usages, and CDR3 sequence identity; (3) identifies the highest expressed alpha chain and beta chain TCR

sequences per droplet; (3) clonality analysis; and (4) all this information is brought together in a meta-data format to include as part of the Seurat analysis, and provides statistics on the number of droplets with heavy and/or light chains and TCR alpha and/or beta chains.

**Differential gene expression analysis and pathway analysis**

Differential gene expression analysis was performed using Seurat's *FindMarkers()* function with the Poisson generalised linear model (GLM). We compared gene expression between two predefined cell clusters or conditions. The Poisson model was selected due to the discrete nature of the single-cell RNA-seq count data and its suitability for low-expression genes. Differentially expressed genes were defined as adjusted p-values <0.05.

The per cell pathway scores for each cell (which quantifies the feature expression programme for each pathway molecule) was calculated using the AddModuleScore using each pathway gene set. The statistics between the levels of each per-cell pathway score between samples was performed using two-sided MANOVA, shown to be statistically significant for each (p-values<1e-10). The mean pathway scores were then determined for each sample for plotting dynamics.

**Public datasets**

Publicly available RNA sequencing datasets for SEM and RS4;11 cell lines were obtained from NCBI (GSE149158)[10] and Cancer Cell Line Encyclopedia (CCLE) DepMap 2019[11] (https://depmap.org/portal).

Transcripts per million (TPM) values for the following 14 genes were extracted from both datasets and collated: PROM1 (CD133), CD19, HLA-A, HLA-B, HLA-C, MICA, MICB, ULBP1, ULBP2, ULBP3, RAET1E (ULBP4), RAET1G (ULBP5) and RAET1L (ULBP6) and CD1D.

For NCBI (GSE149158), GEO2R was used to calculate the normalised TPM for each gene across triplicate samples for SEM (GSM4491229, GSM4491230 and GSM4491231) and RS4;11 (GSM4491211, GSM4491212 and GSM4491213) and the average TPM were subsequently enumerated. For CCLE DepMap, the TPM values for both cell lines were extracted from CCLE 2019 dataset "CCLE RNAseq gene expression data for 1019 cell lines (RSEM, gene)".

**Data availability**

Single cell RNA/TCR datasets have been deposited in Single Cell Portal under the accession: SCP2844, SCP2845, SCP2846, SCP2847, SCP2848 and are available via this link: **(https://singlecell.broadinstitute.org/)**

**Statistics**

Two-tailed Mann-Whitney, Log-rank (Mantel-Cox) tests and ANOVA followed by multiple comparisons testing were used to compare experimental groups as indicated in the figure

legends. Statistical analyses were performed using GraphPad Prism v10. Data are expressed as mean ± SD unless otherwise indicated.

## References (methods only)


1. Roy A, Cowan G, Mead AJ, et al. Perturbation of fetal liver hematopoietic stem and progenitor cell development by trisomy 21. *Proc Natl Acad Sci U S A*. 2012;109(43):17579-17584.
2. Rice S, Jackson T, Crump NT, et al. A human fetal liver-derived infant MLL-AF4 acute lymphoblastic leukemia model reveals a distinct fetal gene expression program. *Nat Commun*. 2021;12(1):6905.
3. Rotolo A, Caputo VS, Holubova M, et al. Enhanced Anti-lymphoma Activity of CAR19-iNKT Cells Underpinned by Dual CD19 and CD1d Targeting. *Cancer Cell*. 2018;34(4):596-610 e511.
4. Rowan AG, Ponnusamy K, Ren H, Taylor GP, Cook LBM, Karadimitris A. CAR-iNKT cells targeting clonal TCRVbeta chains as a precise strategy to treat T cell lymphoma. *Front Immunol*. 2023;14:1118681.
5. Williams MT, Yousafzai Y, Cox C, et al. Interleukin-15 enhances cellular proliferation and upregulates CNS homing molecules in pre-B acute lymphoblastic leukemia. *Blood*. 2014;123(20):3116-3127.
6. Williams MT, Yousafzai YM, Elder A, et al. The ability to cross the blood-cerebrospinal fluid barrier is a generic property of acute lymphoblastic leukemia blasts. *Blood*. 2016;127(16):1998-2006.
7. julian.knight@well.ox.ac.uk CO-M-oBACEa, Consortium CO-M-oBA. A blood atlas of COVID-19 defines hallmarks of disease severity and specificity. *Cell*. 2022;185(5):916-938 e958.
8. Korsunsky I, Millard N, Fan J, et al. Fast, sensitive and accurate integration of single-cell data with Harmony. *Nat Methods*. 2019;16(12):1289-1296.
9. Sivakumar S, Jainarayanan A, Arbe-Barnes E, et al. Single-cell immune multi-omics and repertoire analyses in pancreatic ductal adenocarcinoma reveal differential immunosuppressive mechanisms within different tumour microenvironments. *bioRkiv*. 2023.
10. Pierro J, Saliba J, Narang S, et al. The NSD2 p.E1099K Mutation Is Enriched at Relapse and Confers Drug Resistance in a Cell Context-Dependent Manner in Pediatric Acute Lymphoblastic Leukemia. *Mol Cancer Res*. 2020;18(8):1153-1165.
11. Ghandi M, Huang FW, Jane-Valbuena J, et al. Next-generation characterization of the Cancer Cell Line Encyclopedia. *Nature*. 2019;569(7757):503-508.

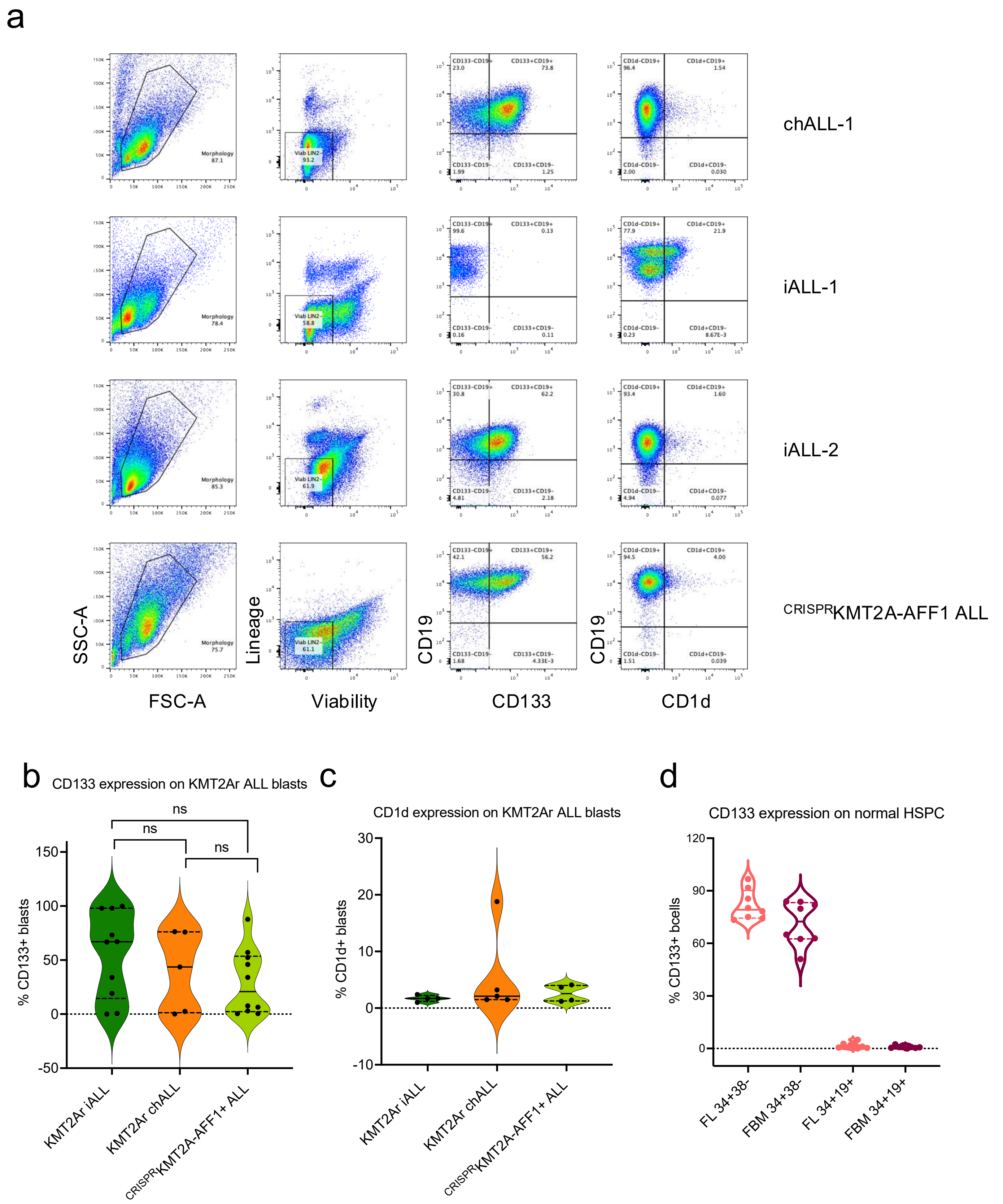


**Suppl Figure 1 related to Fig 1. a.** Representative flow-cytometry plots showing variable CD133 and CD1d co-expression in CD19+ blasts from 3 primary KMT2Ar ALL patient samples (chALL; childhood ALL, iALL: infant ALL) and a human fetal liver (FL) HSPC derived $^{CRISPR}$KMT2A-AFF1 ALL model. **b.** Percentage of CD19+ blasts that express CD133 in KMT2Ar iALL (n=10), KMT2Ar chALL (n=5), and $^{CRISPR}$KMT2A-AF4 ALL (n=10). **c.** Percentage of CD19+ blasts that express CD1d in KMT2Ar iALL (n=4), KMT2Ar chALL (n=5), and $^{CRISPR}$KMT2A-AFF1 ALL (n=4). **d.** Percentage of primitive Lin-CD34+38- progenitors and committed CD34+CD19+ B-progenitors that express CD133 from human fetal liver (FL, n=8) and fetal bone marrow (FBM, n=8).

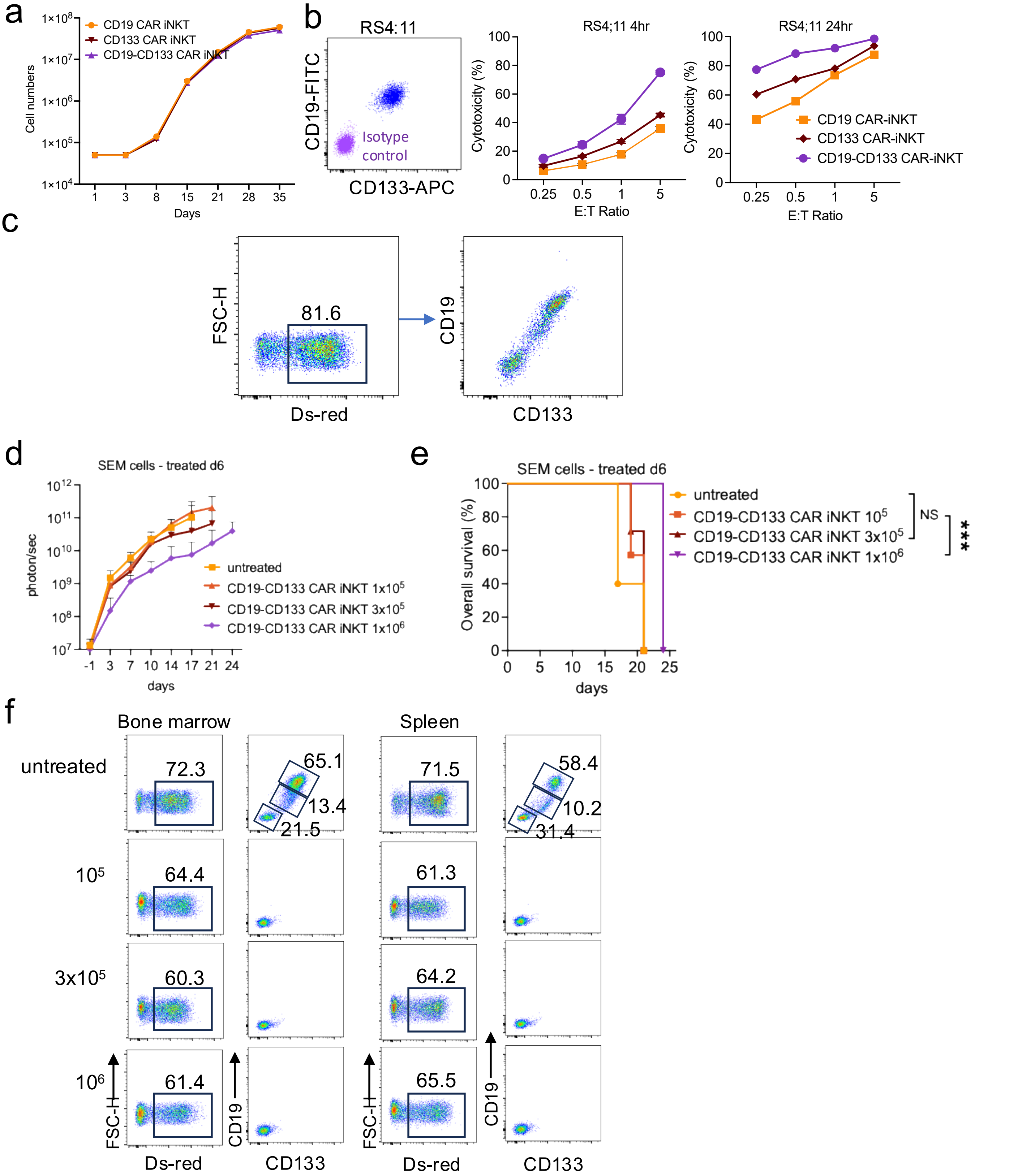


**Suppl Figure 2 related to Fig 1 & 2. a.** Growth curve of CD19, CD133 and CD19-CD133 CAR-iNKT. **b.** Cytotoxicity of mono- and bi-specific CAR-iNKT against the CD19+CD133+ KMT2Ar RS4;11 leukemia cell line (left). Data are from two independent experiments using two different iNKT donors. **c.** Immunophenotypic analysis of an SEM subline propagated in vivo in treatment-free mice. Variable co-expression of CD19 and CD133 in dsRed+ SEM cells. **d & e.** Leukemia burden as assessed by BLI and survival in mice first injected with the SEM subline shown in c followed by treatment with indicated numbers of bi-specific CD19-CD133 CAR-iNKT (n=7 mice per gorup)**.** Kaplan-Meier survival curves analysed by log rank (Mantel-Cox) test adjusted for multiple comparisons. NS, not significant; ***p < 0.001. **f.** Representative flow-cytometric analysis of BM and spleen in sacrificed mice treated with bi-specific CAR-iNKT as shown in d&e.

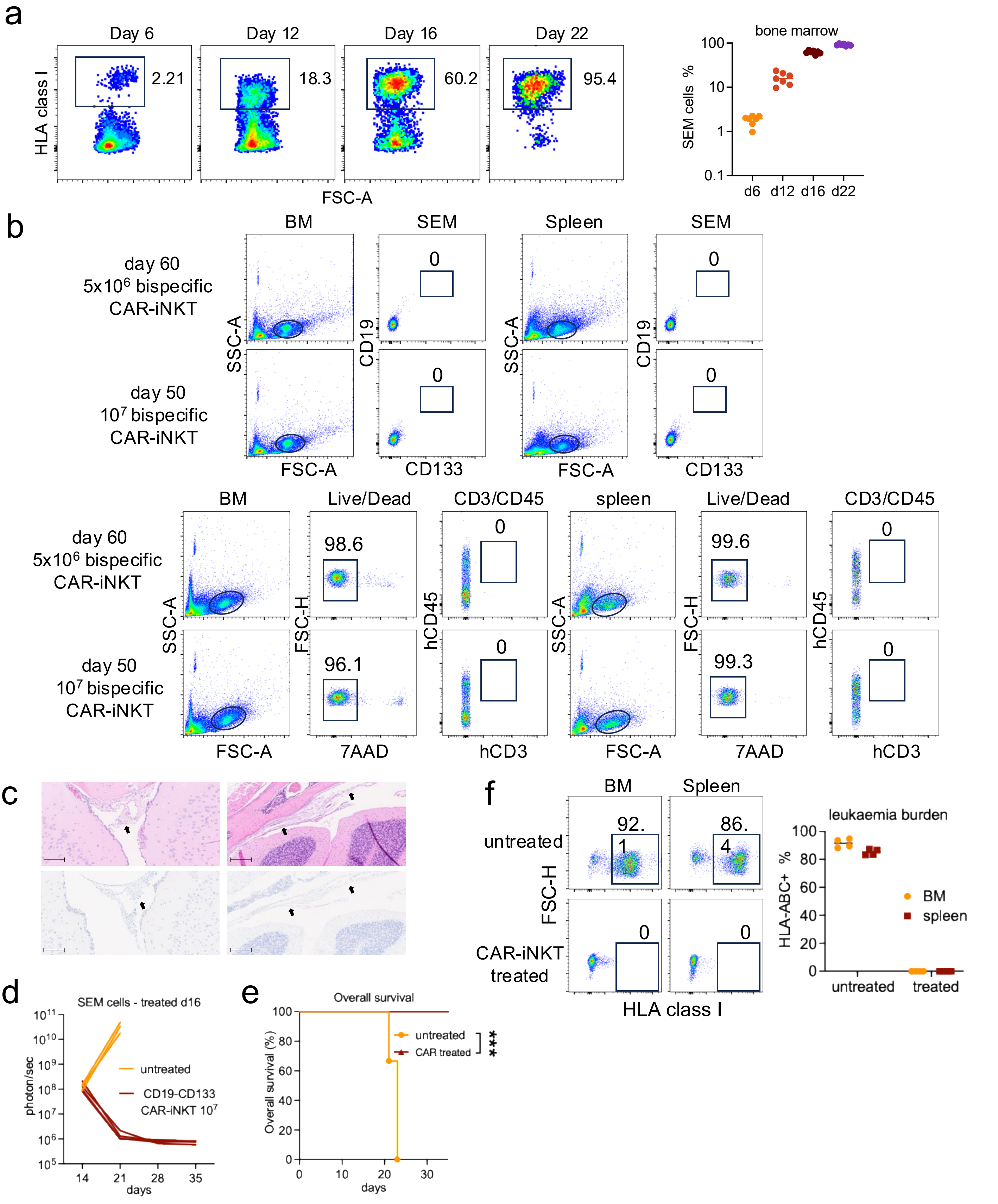


**Suppl Figure 3 related to Fig 2. a.** Flow-cytometric identification of SEM cells in bone marrow after staining with anti-HLA-ABC mAb at different time points following their transfer to NSG mice. Right: cumulative data of leukemia burden in BM on the indicated timepoints (n=7 mice per group). **b.** Flow-cytometric examples for SEM (top) and iNKT (bottom) from both d6 and d12 high dose bispecific CAR-iNKT treated animals culled at day 60 and 50 respectively. **c.** Heads of two bispecific CAR-iNKT-treated animals showing stromal thickening in meninges (arrows), top panel H&E, bottom panel anti human CD19, scale bar 100mm. **d,e.** leukemia burden assessed by BLI and overall survival of mice treated with bi-specific CAR-iNKT on day 16 after leukemia transfer (n=4 mice per group). Kaplan-Meier survival curves of experimental mice over time, $***p < 0.001$, by log rank (Mantel-Cox) test. **f.** Representative flow-cytometric analysis and cumulative data of leukemia burden in bone marrow and spleen of CAR-iNKT-treated and untreated mice as shown in c&d. Leukemia cells are identified as HLA-class I+.

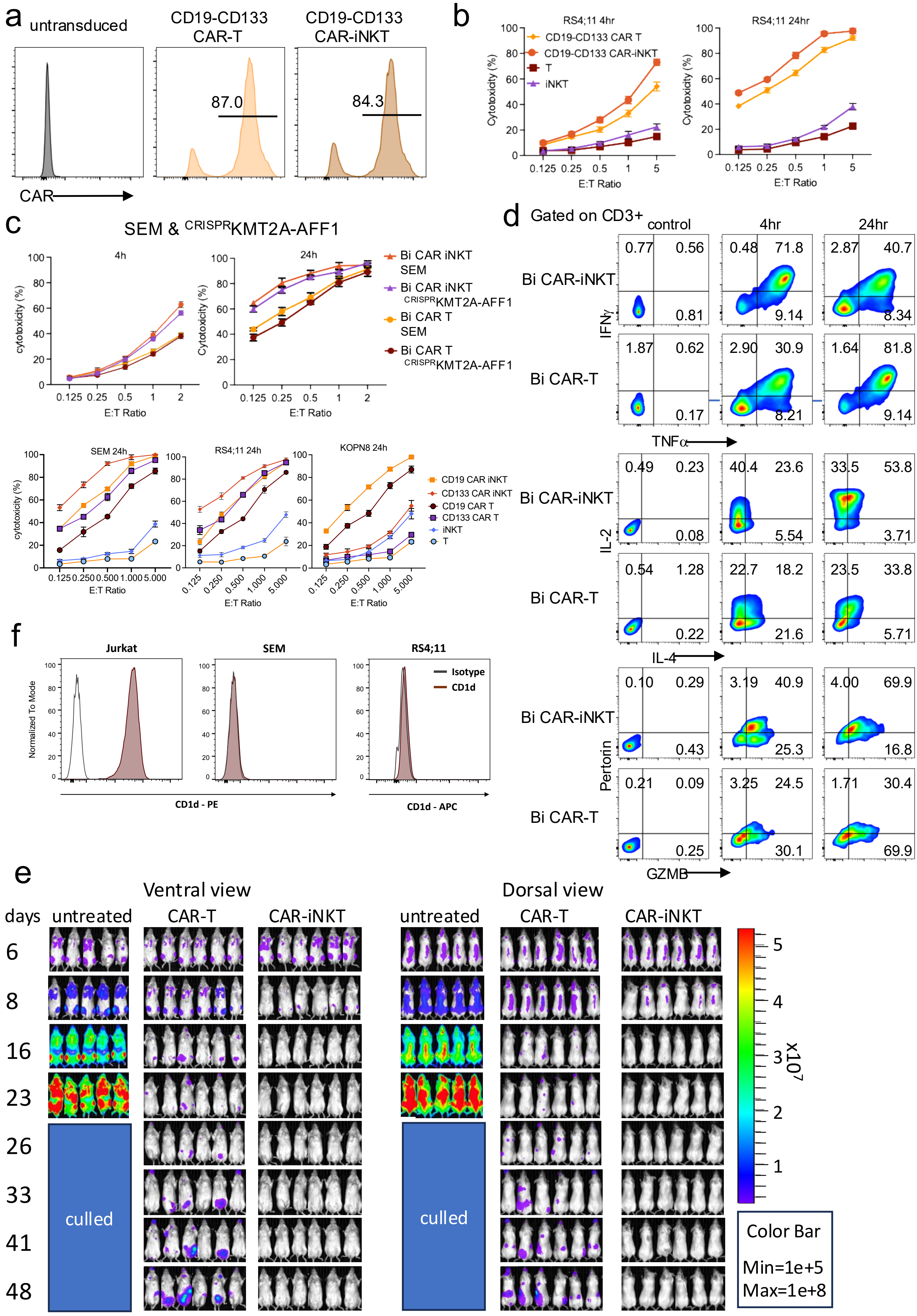

a
untransduced
CD19-CD133 CAR-T
CD19-CD133 CAR-iNKT
87.0
84.3
CAR
b
RS4;11 4hr
RS4;11 24hr
CD19-CD133 CAR T
CD19-CD133 CAR-iNKT
T
iNKT
Cytotoxicity (%)
E:T Ratio
c
SEM & CRISPRKMT2A-AFF1
4h
24h
Bi CAR iNKT SEM
Bi CAR iNKT CRISPRKMT2A-AFF1
Bi CAR T SEM
Bi CAR T CRISPRKMT2A-AFF1
SEM 24h
RS4;11 24h
KOPN8 24h
CD19 CAR iNKT
CD133 CAR iNKT
CD19 CAR T
CD133 CAR T
iNKT
T
d
Gated on CD3+
control
4hr
24hr
Bi CAR-iNKT
Bi CAR-T
IFNγ
TNFα
IL-2
IL-4
Perforin
GZMB
f
Jurkat
SEM
RS4;11
Isotype
CD1d
Normalized To Mode
CD1d - PE
CD1d - APC
e
Ventral view
Dorsal view
days
untreated
CAR-T
CAR-iNKT
6
8
16
23
26
33
41
48
culled
x10^7
Color Bar
Min=1e+5
Max=1e+8

**Suppl Fig 4 related to Fig 4 & 5. a.** Bispecific CAR transduction of T and iNKT cells from the same donor. **b.** Cytotoxic activity at 4 and 24hrs of untransduced T and iNKT and of their bi-specific CAR-transduced counterparts against RS4;11 leukemia cells. **c**. **Top:** 4 and 24hr cytotoxic activity of bi-specific CAR-T and CAR-iNKT against SEM and $^{CRISPR}$KMT2A-AFF1 cells. **Bottom:** CD19 and CD133 mono-specific CAR-transduced and untransduced T and iNKT against the parental CD19+CD133+ SEM and RS4;11 cells and the CD19+CD133- KOPN8 KMT2Ar cells. **d.** Representative flow-cytometric analysis of intracellular cytokine production by CAR-iNKT and CAR-T after their 4 and 24hr co-culture with SEM cells. **e.** BLI images of $5x10^6$ CAR-T- and CAR-iNKT-treated leukemia bearing animals. **f.** CD1d surface expression as assessed by flow-cytometry in SEM and RS4;11 cells. The Jurkat T cell line that expresses CD1d is shown as positive control.

a

**SEM**

TPM

| Gene | GEO (GSE149158) | CCLE DepMap 2019 | |
|---|---|---|---|
| PROM1 (CD133) | 171.6 | 397.7 | SEM key signatures |
| CD19 | 146.4 | 219.2 | |
| HLA-A | 212.1 | 247.1 | MHC Class 1 |
| HLA-B | 406.2 | 448.2 | |
| HLA-C | 229.0 | 264.6 | |
| MICA | 8.7 | 11.6 | NKG2D ligands |
| MICB | 23.6 | 26.3 | |
| ULBP1 | 1.0 | 1.6 | |
| ULBP2 | 1.9 | 1.4 | |
| ULBP3 | 4.0 | 1.7 | |
| RAET1E (ULBP4) | 0.0 | 0.0 | |
| RAET1G (ULBP5) | 0.0 | 0.0 | |
| RAET1L (ULBP6) | 0.2 | 0.0 | |
| CD1D | 0.1 | 0.2 | |

**RS4;11**

TPM

| Gene | GEO (GSE149158) | CCLE DepMap 2019 | |
|---|---|---|---|
| PROM1 (CD133) | 124.5 | 136.0 | RS4;11 key signatures |
| CD19 | 84.9 | 133.3 | |
| HLA-A | 305.0 | 274.1 | MHC Class 1 |
| HLA-B | 534.3 | 471.7 | |
| HLA-C | 195.1 | 169.4 | |
| MICA | 7.4 | 7.0 | NKG2D ligands |
| MICB | 26.0 | 5.3 | |
| ULBP1 | 0.0 | 0.0 | |
| ULBP2 | 0.4 | 0.3 | |
| ULBP3 | 0.5 | 0.0 | |
| RAET1E (ULBP4) | 0.0 | 0.0 | |
| RAET1G (ULBP5) | 0.0 | 0.0 | |
| RAET1L (ULBP6) | 0.1 | 0.0 | |
| CD1D | 0.0 | 0.0 | |

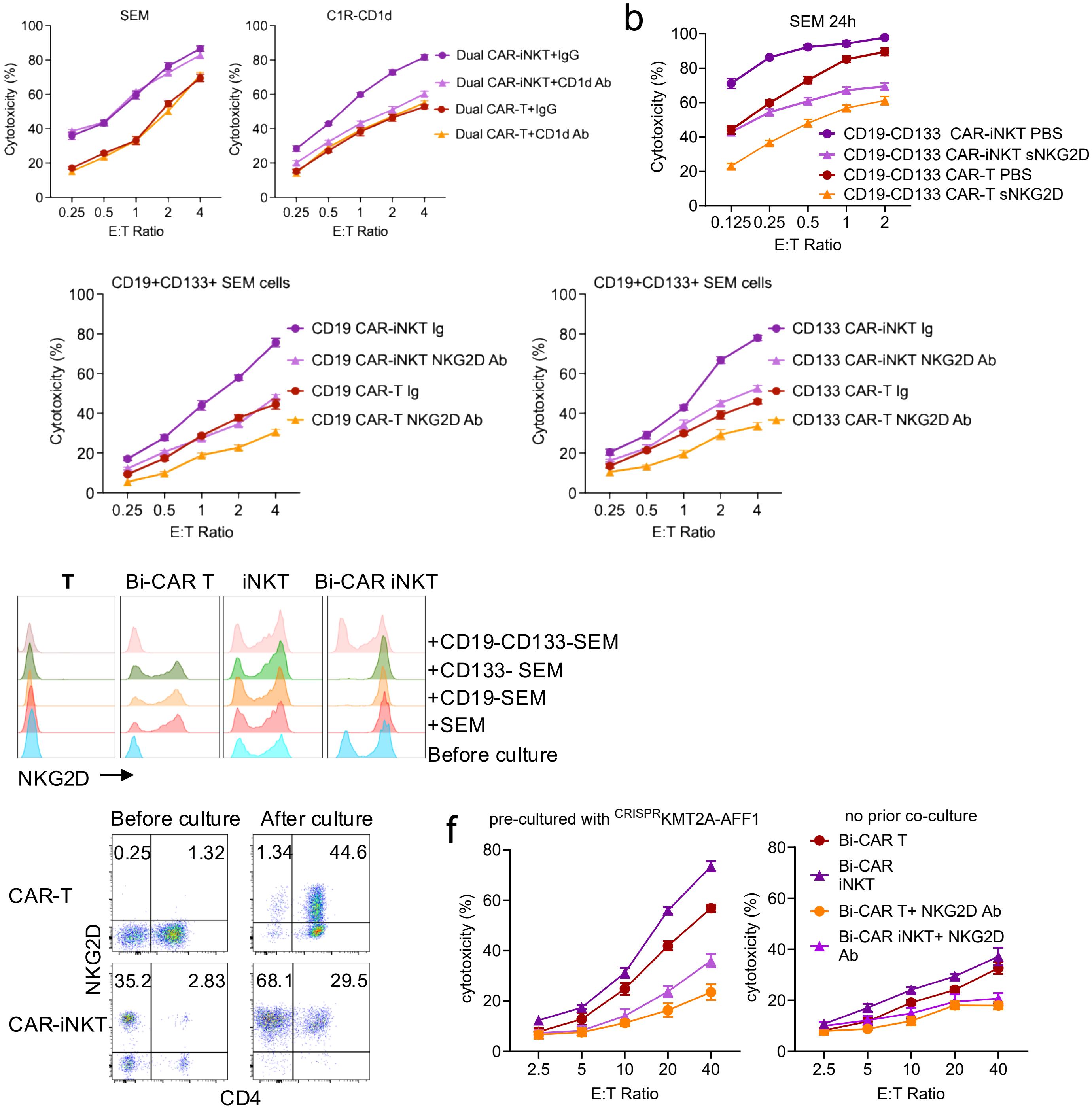

**Suppl Figure 5 related to Fig 5. a. Top:** mRNA expression of NKG2D ligands in SEM and RS4;11 cells as assessed by RNA-seq**. Bottom:** Cytotoxicity assay of bi-specific CAR-iNKT against SEM and the CD19+CD1d+ C1R-CD1d cells in the presence of anti-CD1d or Ig isotype control (5μg/ml). **b.** 24hr cytotoxicity of bi-specific CAR-T and-iNKT that had been pre-cultured with SEM cells against SEM cells in the presence of 10μg of NKG2D-Fc protein or PBS control. **c**. Mono-specific CAR-iNKT and CAR-T and their cytotoxic activity against SEM cells in the presence of NKG2D blocking Ab or isotype control. **d.** Representative examples of the FACS analysis of data shown in Fig 4h. **e.** Representative flow-cytometric examples of NKG2D expression on CAR-iNKT/T pre-cultured with parental and gene-edited SEM cells as shown in Fig 4f&h. **e.** Upregulation of NKG2D in CAR-T vs CAR-iNKT after 24hr co-culture with $^{CRISPR}$KMT2A-AFF1 leukemia cell. **f.** Left: Bi-specific CAR-iNKT pre-cultured with $^{CRISPR}$KMT2A-AFF1 leukemia cells are subsequently more cytotoxic than CAR-T against **C**AR target-negative CD19-CD133-SEM cells in an NKG2D-dependent manner. Right: Cytotoxicity of CAR-iNKT/T that had not been pre-cultured with $^{CRISPR}$KMT2A-AFF1. Representative of two independent experiments using two different iNKT donors.

a

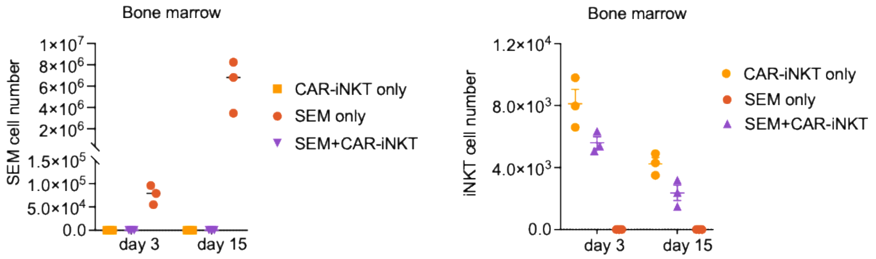


b

| | Total cells with GEX | TCR_status: TRA+ | TCR_status: TRA+TRB+ | TCR_status: TRB+ | TCR_status: none |
|---|---|---|---|---|---|
| BMd15_CAR_7489cells | 1526 | 94 | 472 | 138 | 822 |
| BMd15_CAR_SEM_2768 | 345 | 22 | 166 | 16 | 141 |
| BMd3_CAR_6349cells | 1115 | 55 | 565 | 126 | 369 |
| BMd3_CAR_SEM_7645 | 2141 | 124 | 964 | 275 | 778 |
| Naive_CAR_30100 | 8426 | 1244 | 5514 | 708 | 960 |

c

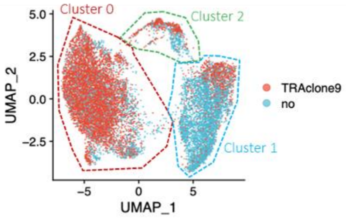


d

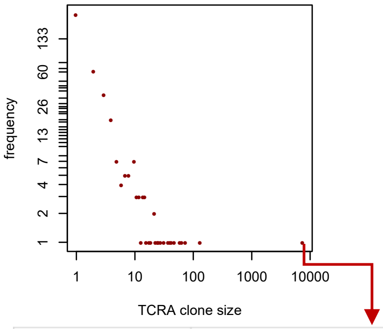


| | |
|---|---|
| TRA clone ID | Clone 9 |
| TRA V gene | TRAV10*01 |
| TRA J gene | TRAJ18*01 |
| CDR3 regions (amino acid) | CVVSDRGSTLGRLYF |
| CDR3 regions (nucleotide) | TGTGTGGTGAGCGACAGAGGCTCAACCCTGGGGAGGCTATACTTT |

e

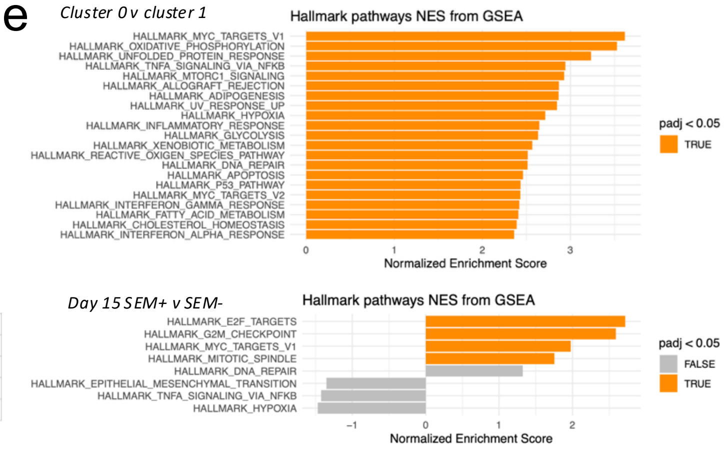


f

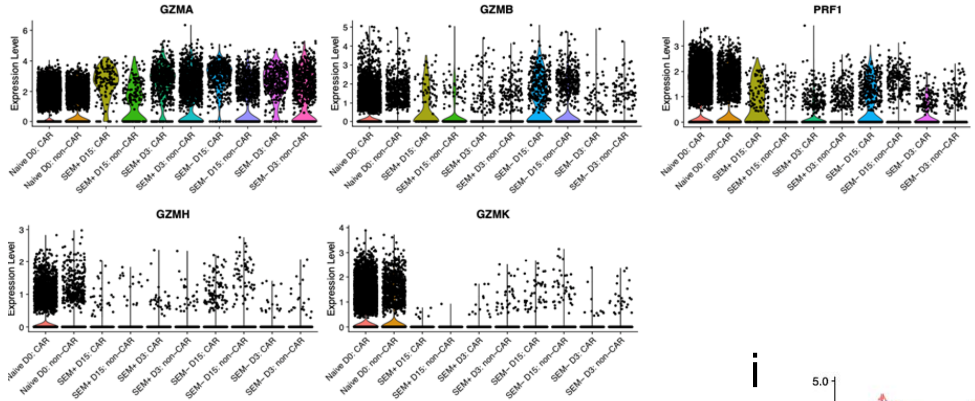


g

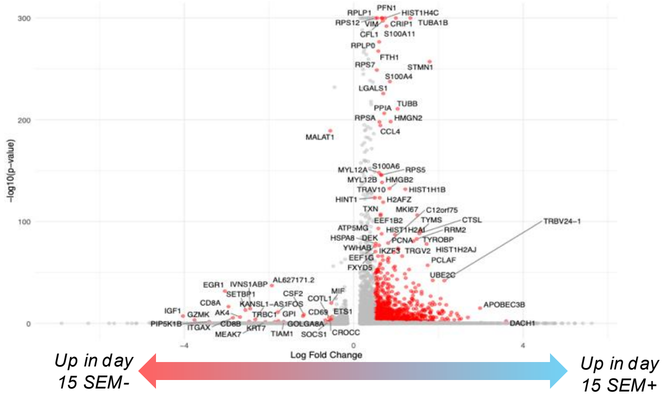


i

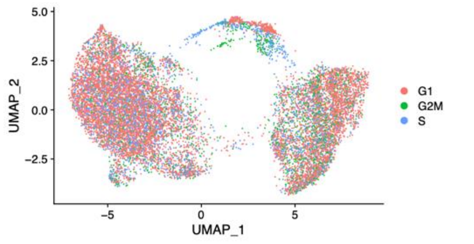


| Number of cells (%) | Cluster 0 | Cluster 1 | Cluster 2 |
|---|---|---|---|
| Non-S phase | 6101 (75.4%) | 3677 (78.6%) | 486 (62.1%) |
| S phase | 1992 (24.6%) | 1001 (21.4%) | 296 (37.9%) |

h

| Sample | Number (%) of cells in non-S phase | Number (%) of cells in S phase | p-value (between SEM+/-) |
|---|---|---|---|
| BMd3_CAR_SEM_7645 | 1541 (72%) | 600 (28%) | 0.3268 |
| BMd3_CAR_6349cells | 784 (70.3%) | 331 (29.7%) | |
| BMd15_CAR_SEM_2768 | 195 (56.5%) | 150 (43.5%) | 6.20E-06 |
| BMd15_CAR_7489cells | 1060 (69.5%) | 466 (30.5%) | |

**Suppl Fig 6 related to Fig 6.**

**Suppl Figure 6 related to Fig 6. a.** Numbers of iNKT and SEM cells in the bone marrow of mice receiving bi-specific CAR-iNKT only or SEM leukemia cells and CAR-iNKT. **b.** Table showing the number of cells in which TCR transcripts were identified. **c.** Overlay of TCR (TRA)- expressing cells on the UMAP map. **d.** Clone size of the invariant TCRVa24Ja18 clonotype. **e.** GSEA of genes over-expressed in (top) clusters 0 & 1 and (bottom) between cells isolated from the bone marrow of leukemia-bearing- vs leukemia-free mice on day 15, with reference to MSigDB Hallmarks genesets. **f.** Relative expression of indicated genes in the different experimental subgroups**. g.** Volcano plot showing differential gene expression between CAR-iNKT cells isolated from the bone marrow of leukemia-bearing- vs leukemia-free mice on day 15 (log2FC>1 and padj< 0.05). **h.** Frequency of S phase in CAR-iNKT cells from leukemia-bearing and -free mice on day 3 and day 15. Fisher exact test. **I.** Projection of cell cycle related signatures on UMAP. Cluster 2 is the most enriched for proliferative cells (that cluster 2 is more proliferative than clusters 0 and 1. P-value= 2.894e-18, Fisher exact test).

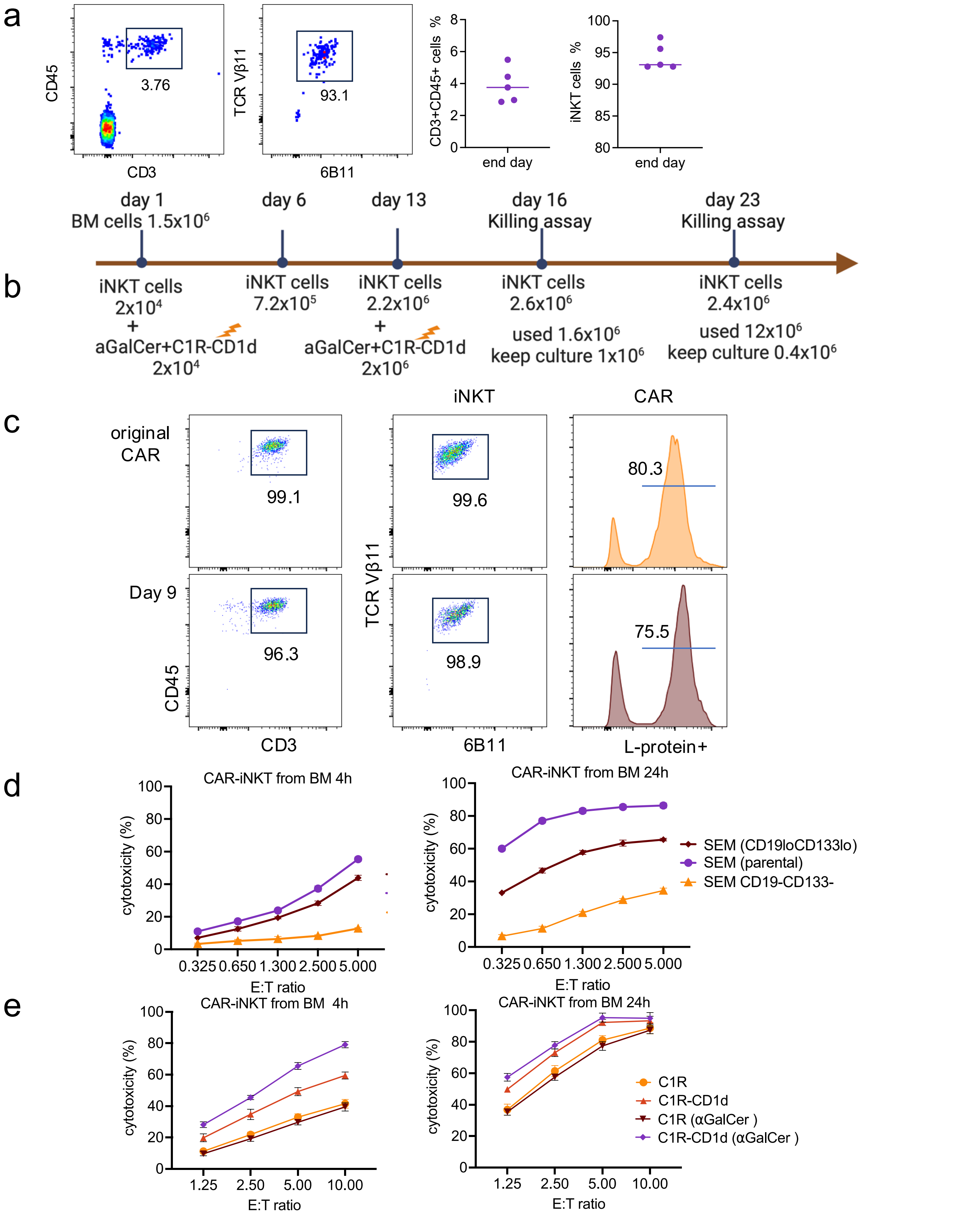


**Suppl Figure 7 related to Fig 6. a.** Left: Flow-cytometric identification of iNKT cells in the BM at sacrifice of animals described in Fig 2a&b. Right: cumulative data for a. **b.** Schematic of expansion and functional analysis of iNKT cells form a. **c.** Purity and CAR expression by iNKT of day 9 post ex vivo selection and expansion. **d.** Cytotoxic activity at 4 and 24hr of day 16 ex vivo expanded CAR-iNKT against parental SEM, SEM with variable co-expression of CD19 and CD133 (BM; Suppl Fig 2d) and gene-edited SEM cells lacking expression of CD19 and CD133. **e.** 4 and 24hr cytotoxicity assay with day 23 CAR-iNKT against the CD19+CD133- C1R and C1R-CD1d cells in the presence or not of aGalCer (100ng/ml). Representative of two independent experiments.

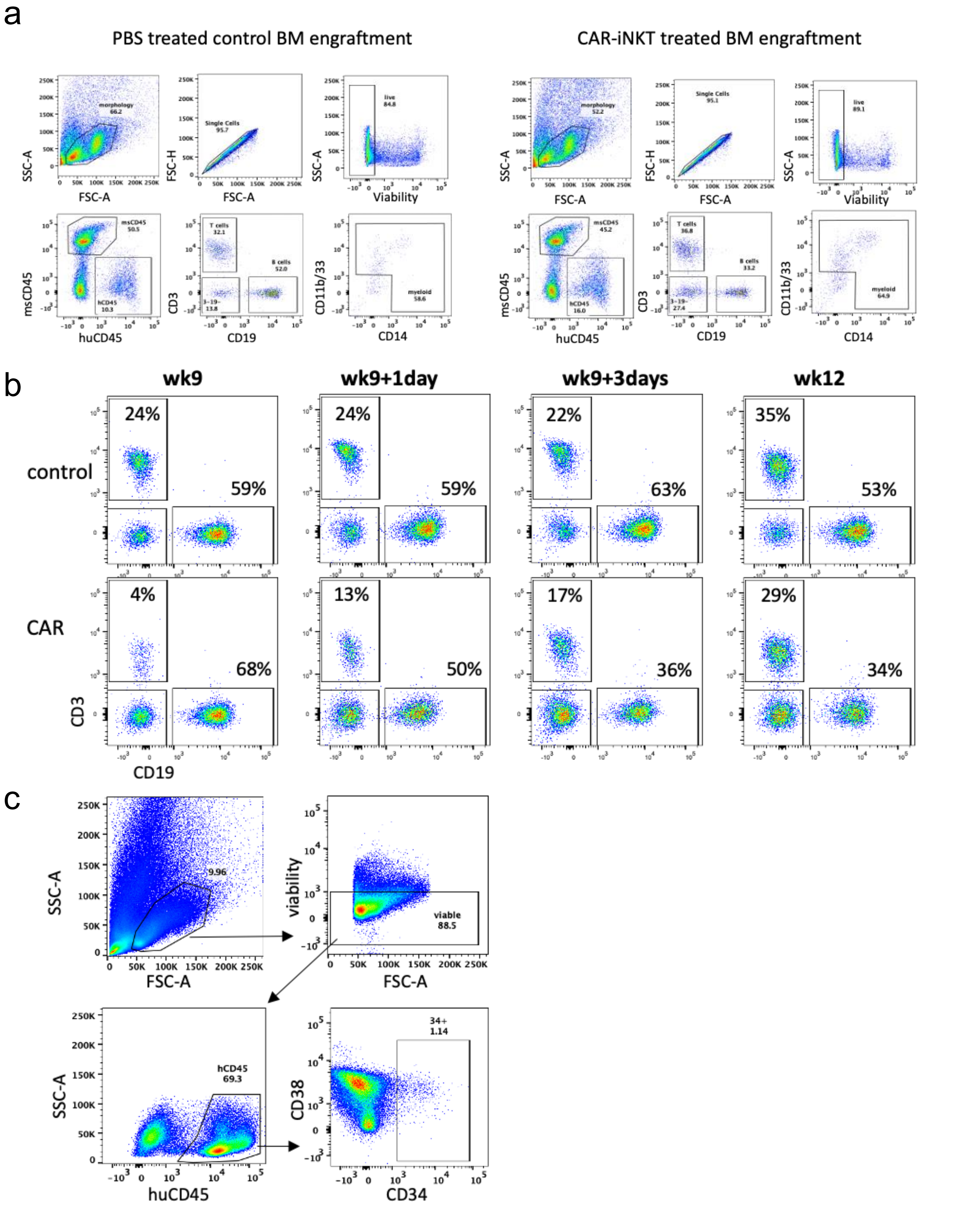


**Suppl Figure 8 related to Fig 7. Analysis of peripheral blood and bone marrow of humanized mice receiving bi-specific CAR-iNKT. a.** representative flow plots showing gating strategy used to determine peripheral blood (PB) and bone marrow (BM) engraftment and lineage output. The data shown is from BM at cull for PBS treated (left) and CAR-iNKT treated (right) mice. **b.** Representative flow plots showing frequency of CD19+ B cells and CD3+ T cells in the PB of control mice treated with PBS (top row) and mice treated with CAR-iNKT at 9 weeks (bottom row). Data shown as % of hCD45+ cells. **c.** Representative flow plots showing gating strategy used to determine immature CD34+ cells in the BM at cull.

**Suppl Table 3:** Guides for CRISPR knockout

| | |
|---|---|
| CD19-28932346 | CACAGCGUUAUCUCCCUCUG |
| CD19+28932373 | CGCUGUGCUGCAGUGCCUCA |
| PROM1-16075877 | UCGGCUCCCUGUUGCUGCUG |
| PROM1+16075896 | GCAACAGGGAGCCGAGUACG |
| PROM1-16075896 | CUAGCUAUGGCCCUCGUACU |

| Anitbody | Fluorophore | Source | Cat. No. | clone |
|---|---|---|---|---|
| TCR Va24-Ja18 | BV421 | Biolegend | 342916 | 6B11 |
| FMC63 CD19-CAR Idioty | APC | Miltenyi | 130-127-343 | FMC63 |
| CD56 | BV605 | Biolegend | 318334 | HCD56 |
| CD56 | FITC | Life Tech | 11-0566-42 | HCD56 |
| L-Protein | PE | Stratech(SinoBiologic | 11044-H07E-I | RecProtein |
| Viability | 7AAD | Cayman | 11397 | |
| mCD45.1 | APCcy7 | Biolegend | 103116 | 30-F11 |
| CD45 | af700 | Life Tech | 56-9459-42 | 2D1 |
| CD20 | ef450 | Life Tech | 48-0209-42 | 2H7 |
| CD16 | PerCPcy5.5 | Biolegend | 302028 | 3G8 |
| CD34 | PEcy7 | Life Tech | 25-0349-42 | 4H11 |
| CD133/1 | APC | Miltenyi Biotec | 130-113-106 | AC133 |
| CD133 | PE | Miltenyi | 130-113-108 | AC133 |
| CD10 | FITC | Life Tech | 11-0106-42 | eBioCB-CALL |
| CD56 | PerCPcy5.5 | Biolegend | 318322 | HCD56 |
| CD19 | APC | Biolegend | 302212 | HIB19 |
| CD19 | PEcy7 | Life Tech | 25-0199-42 | HIB19 |
| CD19 | FITC | eBioscience | 11-0199-42 | REA675 |
| CD235a | PerCPcy5.5 | Biolegend | 306614 | HIR2 |
| CD38 | BV605 | Biolegend | 303532 | HIT2 |
| CD14 | PerCPcy5.5 | Biolegend | 301824 | M5E2 |
| CD3 | af700 | Life Tech | 56-0037-42 | okt3 |
| CD3 | BV711 | Biolegend | 317328 | OKT3 |
| CD3 | PerCPcy5.5 | Biolegend | 317336 | OKT3 |
| CD2 | beads | Miltenyi | 130-091-114 | |
| CD2 | PerCPcy5.5 | Biolegend | 300216 | RPA-2.10 |
| Viability | Hoescht58 | Life Tech | H3569 | |
| Viability | ef506 | Life Tech | 65-0866-18 | |
| CD1d | PE | BD Pharmingen™ | 550255 | CD1d42 |
| CD1d | PE | Biolegend | 350306 | 51.1 |
| CD1d | BB790 | BD Bioscience | custom | |
| CD45 | FITC | BD Bioscience | 345808 | 2D1 |
| mCD45 | APCcy7 | Biolegend | 103116 | 30-F11 |
| TCR Va24-Ja18 (iNKT ce | BV421 | Biolegend | 342916 | 6B11 |
| Viability | 7AAD | Cayman | 11397 | |
| Labeling Check Reagent | APC | Miltenyi | 130-122-219 | |
| CD11b | FITC | Biolegend | 301330 | ICRF44 |
| CD33 | FITC | Biolegend | 366620 | P67.6 |
| Brilliant stain buffer plus | | BD Bioscience | 566385 | |
| CD90 | BV421 | Biolegend | 328122 | 5E10 |
| CD16 | PerCPcy5.5 | Biolegend | 302028 | 3G8 |
| CD34 | PEcy7 | Life Tech | 25-0349-42 | 4H11 |
| CD123 | BV650 | Biolegend | 306020 | 6H6 |
| CD133 | PE | Miltenyi | 130-113-108 | AC133 |
| CD10 | FITC | Life Tech | 11-0106-42 | eBioCB-CALL |
| CD45 | APC/Cyanine | BioLegend | 103116 | 30-F11 |
| CD45 | Brilliant Violet | BioLegend | 368526 | 2D1 |

| | | | | |
|---|---|---|---|---|
| CD45RA | APCef780 | Life Tech | 47-0458-42 | HI100 |
| CD45 | BUV395 | BD Bioscience | 563792 | HI30 |
| CD235 | PerCP cy5.5 | Biolegend | 306614 | HIR2 |
| CD38 | af700 | Life Tech | 56-0389-42 | HIT2 |
| Brilliant stain buffer plus | | BD Bioscience | 566385 | |
| CD4 | Brilliant Ultra ' | eBioscience | 363-0042-80 | RM4-5 |
| CD4 | PE | BioLegend | 317410 | OKT4 |
| CD4 | PE | BioLegend | 300539 | RPA-T4 |
| CD8a | FITC | BioLegend | 301005 | RPA-T8 |
| CD8 | FITC | BioLegend | 344704 | SK1 |
| 7-AAD | Viability Staini | BioLegend | 420404 | |
| TCR Vb11 | APC | Miltenyi Biotec | 130-125-508 | REA559 |
| Protein L | PE | Stratech Scientific | 11044-H07E-P | |
| HLA-A,B,C | Pacific Blue™ | BioLegend | 311418 | W6/32 |
| CD3 | APC/Fire™ 7ℇ | BioLegend | 300470 | UCHT1 |
| NKG2D Fc | | Bio-Techne | 1299-NK-050 | |
| CD314 (NKG2D) | | eBioscience | 16-5878-82 | 1D11 |
| iNKT | MicroBeads | Miltenyi Biotec | 130-094-842 | |
| iNKT | APC | Miltenyi Biotec | 130-094-839 | |
| EasySep™ Release Human PE Positiv | | STEMCELL Technologies | | |
| Granzyme B | PE | BioLegend | 372207 | QA16A02 |
| Granzyme B | PE-eFluor 610 | eBioscience | 61-8898-82 | gb11 |
| IFN-gamma | Brilliant Violet | BioLegend | 502544 | 4S.B3 |
| IFN gamma | Alexa Fluor 7( | eBioscience | 56-7319-42 | 4S.B3 |
| TNF-alpha | Brilliant Violet | BioLegend | 502940 | MAb11 |
| Perforin | FITC | eBioscience | 11-9994-42 | dG9 |
| IL-4 | PE-Cyanine7 | eBioscience | 25-7049-41 | 8D4-8 |
| IL-2 | FITC | BioLegend | 500304 | MQ1-17H12 |
| CD3 | MicroBeads | | 130-097-043 | OKT3 |
| CD28 | MicroBeads | | 130-093-375 | |
